# 2-Bromopyridines as Versatile Synthons for Heteroarylated 2-Pyridones via Ru(II)-Mediated Domino C–O/C–N/C–C Bond Formation Reactions

**DOI:** 10.3390/molecules29184418

**Published:** 2024-09-17

**Authors:** Miha Drev, Helena Brodnik, Uroš Grošelj, Franc Perdih, Jurij Svete, Bogdan Štefane, Franc Požgan

**Affiliations:** Faculty of Chemistry and Chemical Technology, University of Ljubljana, Večna pot 113, 1000 Ljubljana, Sloveniafranc.perdih@fkkt.uni-lj.si (F.P.); jurij.svete@fkkt.uni-lj.si (J.S.);

**Keywords:** ruthenium catalysis, heterocycles, C–H activation, pyridones, 2-bromopyridines

## Abstract

A novel methodology for the synthesis of 2-pyridones bearing a 2-pyridyl group on nitrogen and carbon atoms, starting from 2-bromopyridines, was developed employing a simple Ru(II)–KOPiv–Na_2_CO_3_ catalytic system. Unsubstituted 2-bromopyridine was successfully converted to the penta-heteroarylated 2-pyridone product using this method. Preliminary mechanistic studies revealed a possible synthetic pathway leading to the multi-heteroarylated 2-pyridone products, involving consecutive oxygen incorporation, a Buchwald–Hartwig-type reaction, and C–H bond activation.

## 1. Introduction

**Scheme 1:**
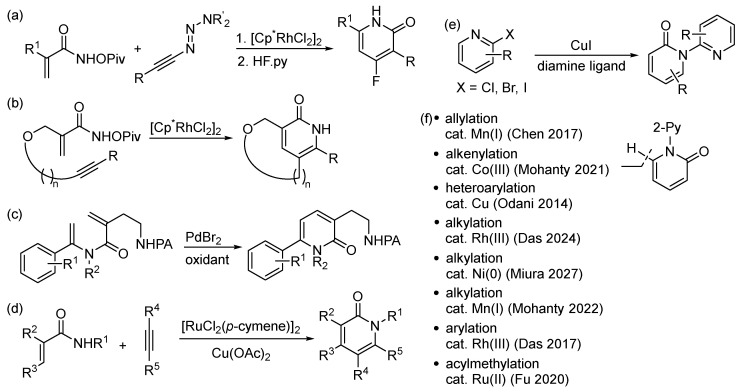
Content status: selected syntheses of 2-pyridones (**a**–**e**) and C–C bond-forming reactions through C6–H functionalization of a 2-pyridone ring (**f**) [34,37,38,39,40,41,42,45].

Pyridine derivatives and related azaheterocycles are widely found as a central structural motif in biologically active compounds, natural products [1,2,3,4,5], and functional materials [6]. Furthermore, pyridine-based ligands [7] are characterized by high stability and tolerance to fluctuating redox environments, and are frequently used for the construction of metallosupramolecular architectures [8,9], as well as in crucial catalytic transformations [10]. It has been demonstrated that the 2-pyridone ring is an important building block [11,12,13] for the construction of valuable nitrogen-containing heterocyclic systems [14]. In addition, 2-pyridone ligands substituted with electron-withdrawing groups have been identified to facilitate site-selective C(*sp*^3^)–H fluorination [15] and arylation [16,17]. Therefore, there is a strong interest in synthetic, and especially catalytic, methods for the preparation [18] and functionalization [19] of 2-pyridones. For example, 3,6-disubstituted 4-fluoro-2-pyridones were prepared by Rh-catalyzed C–H activation–Lossen rearrangement followed by a Wallach reaction starting from alkynyl triazenes and *N*-(pivaloyloxy)acrylamides in a one-pot procedure (Scheme 1a) [20]. Rh-catalyzed C–H activation and intramolecular heterocyclization of ω-alkynyl α-substituted hydroxamates led to macrocyclic 2-pyridones (Scheme 1b) [21]. A 1,3,6-trisubstituted 2-pyridone scaffold was obtained by C–H/C–H coupling of *N*-alkenyl acrylamides exposed to a Pd(II) catalyst together with an oxidizing agent (Scheme 1c) [22]. Ackermann’s group introduced the Ru(II)-catalyzed oxidative annulation reaction of alkynes by acrylamides to form substituted 2-pyridones by N–H/C–H activations (Scheme 1d) [23]. The incorporation of various functional groups directly into the 2-pyridone core was successfully achieved by applying catalytic C–H bond functionalization strategies [24,25]. In this context, the 2-pyridone moiety was extensively explored for selective C-3 [26,27,28,29], C-4 [30], and C-5 [31,32,33] direct functionalization under transition metal catalyzed conditions. On the other hand, the selective functionalization of the more electron-deficient C-6 position of *N*-heteroaryl-pyridin-2-ones was mainly controlled by the 2-pyridyl substituent [34,35,36,37]. Miura and co-workers reported Cu-catalyzed dehydrogenative heteroarylation at the C-6 position with 1,3-azoles [38]. Several groups reported rhodium- [39], nickel- [40], or manganese-catalyzed [41] C-6-alkylation of 2-pyridones [42]. Miura and co-workers investigated C-6-borylation [43] catalyzed by rhodium complexes, while Liu’s research group reported C-6-arylation of 2-pyridones [44]. Wang and co-workers described a synthetic method for C-6-acylmethylation of *N*-pyridyl-2-pyridones catalyzed by the Ru(II) complex [45]. However, the direct catalytic C-6-heteroarylation of 2-pyridone using stable Ru(II) catalysts remains practically unexplored.

## 2. Results and Discussion

**Scheme 2:**
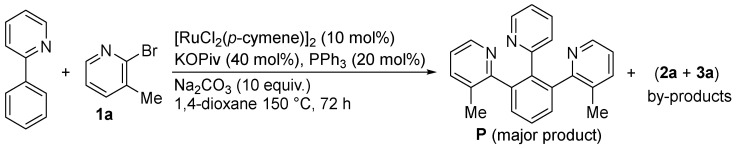
The formation of pyridone by-products **2a** + **3a** in C–H-heteroarylation of 2-pyridylbenzene (our previous work [46]).

Recently we attempted the synthesis of hexaheteroarylbenzenes by applying Ru(II)-catalyzed multiple C–H functionalization of benzene substrates with pyridyl, pyrimidyl, or pyrazolyl directing groups [46]. Interestingly, in the case of 2-pyridylbenzene as the substrate and 3-methyl-2-bromopyridine (**1a**) as the arylating agent, di-*ortho*-heteroarylated 2-pyridylbenzene **P** was isolated as the major product together with 2-pyridone by-products **2a** and **3a** (Scheme 2). While we could not conclusively distinguish the N-arylated from the also possible O-arylated product by NMR spectroscopy, X-ray analysis of the self-condensation product **3a** unambiguously revealed that one pyridyl group is attached to the nitrogen atom of the 2-pyridone ring, while the second is bonded to the C-6 carbon atom (see ESI, Figure S1). To our best knowledge, there is only one report of the direct conversion of 2-halopyridines to *N*-(2-pyridyl)pyridin-2-ones, which was achieved by using a CuI-trans-*N,N’*-dimethylcyclohexane-1,2-diamine-K_2_CO_3_ catalyst system (Scheme 1e) [47]. The authors hypothesized that 2-bromopyridine hydrolyzes under the established reaction conditions and undergoes a coupling reaction with non-hydrolyzed 2-bromopyridine, but the reaction pathway was not examined.

In this article, we report the optimization process of the formation of 2-pyridone derivatives directly from 2-bromo-3-methylpyridine in the presence of a Ru(II) catalyst. Based on our preliminary study, a plausible mechanistic route for the formation of *N*-pyridyl-2-pyridone is also suggested.

We initiated optimization studies by subjecting 2-bromo-3-methylpyridine (**1a**) to similar reaction conditions as we previously described for multiple heteroarylaton of 2-phenylpyridine [46]. Pleasingly, after 3 days, pyridones **2a** and **3a** were obtained in 40% and 35% relative yields, respectively, together with 25% of the remaining 2-bromopyridine **1a**, as revealed by NMR analysis of the crude reaction mixture (Table 1, entry 1). As expected, no conversion of pyridine **1a** was observed without catalyst addition. Next, we investigated which additives are needed for the efficient formation of pyridone **3a**, since heating **1a** together with the catalyst [RuCl_2_(*p*-cymene)]_2_ alone also did not lead to product formation. When either KOPiv or Na_2_CO_3_ were used together with 1 mol% of [RuCl_2_(*p*-cymene)]_2_, only a small quantity of 2-pyridone products was formed (Table 1, entries 2 and 3). We hypothesized that both a base and ligand must cooperate for a smooth reaction to occur. Indeed, the addition of 4 mol% of KOPiv and 1.25 equivalents of Na_2_CO_3_ gave a quantitative **1a** conversion with a ratio of 80/20 of the products **2a**/**3a** (Table 1, entry 4). To our delight, increasing the amount of Ru(II) dimer (5 mol%) and KOPiv (20 mol%) resulted in a complete conversion of **1a** to a mixture of **2a** and **3a**, with 2-pyridone **3a** being the major product (Table 1, entry 5). We concluded that the interaction of the Ru(II) center with the Na_2_CO_3_–KOPiv system is crucial for the efficient formation of *N*-pyridylpyridin-2-one. With the optimal catalyst system in hand, we further focused on evaluating the solvent effect. A significant reduction in the formation of pyridone **3a** was observed in *N*-methyl-2-pyrrolidone (NMP), 2-methyltetrahydrofuran (2-MeTHF), and diethyl carbonate (DEC) (Table 1, entries 6–8). A quantitative conversion with the highest relative amount of product **3a** was achieved when the reaction was carried out in toluene (Table 1, entry 9). Interestingly, the reaction was completely inhibited in water, while the mixture of 1,4-dioxane/water allowed a 25% conversion of **1a** (Table 1, entries 10 and 11).

**Scheme 3:**
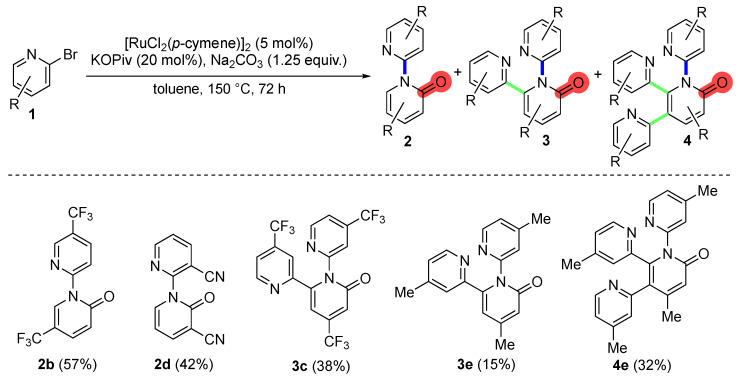
The scope of the *N*-pyridyl-pyridin-2-ones formation.

The extent of catalytic transformation was investigated with a rather small group of 2-bromopyridines **1** under the optimized reaction conditions ([RuCl_2_(*p*-cymene)]_2_ (5 mmol%), KOPiv (20 mol%), Na_2_CO_3_ (1.25 equiv.), toluene, 150 °C, 72 h) (Scheme 3). From the reaction of 2-bromo-5-trifluoromethylpyridine, which contains an electron-withdrawing group at position C-5, we were only able to isolate the monopyridyl substituted 2-pyridone **2b** in a 57% yield. Similarly, the reaction of 2-bromo-4-trifluoromethylpyridine containing a CF_3_ group at the C-4 position gave quantitatively only the pyridyl-disubstituted 2-pyridone **3c** in an isolated yield of 38%, while 2-bromo-3-cyanopyridine bearing an electron-withdrawing group at the C-3 position formed exclusively pyridone **2d** (42% isolated yield). In addition, 2-bromo-4-methylpyridine with an electron-donating methyl group at the C-4 position converted almost quantitatively into a mixture of the 2-pyridones **3e** and **4e** with 15% and 32% isolated yields, respectively. In contrast, 2-bromopyridines with an electron-donating group at the C-3 (2-bromo-3-methoxypyridine), C-5 (2-bromo-5-methoxypyridine and 2-bromo-5-methylpyridine) or C-6 position (2-bromo-6-methylpyridine) did not react under the given reaction conditions.

**Scheme 4:**
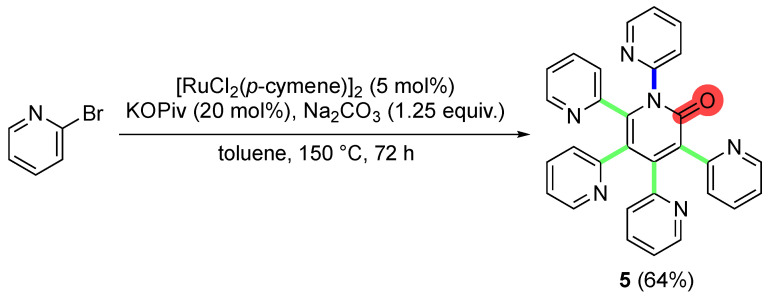
The synthesis of polypyridyl-2-pyridone **5**.

We were pleased to obtain the fully heteroarylated 2-pyridone **5** (64% isolated yield) directly from 2-bromopyridine by applying the optimized reaction conditions (Scheme 4).

**Scheme 5:**
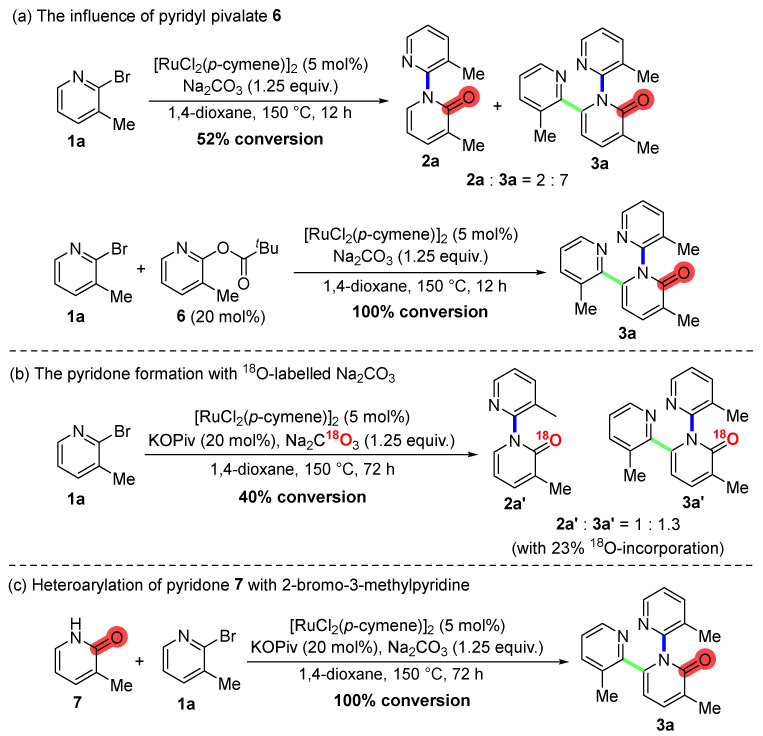
Control experiments.

A series of experiments was performed to gain insight into the mechanism of 2-pyridone formation (Scheme 5). First, we carried out the reaction of 2-bromo-3-methylpyridine (**1a**) with 10 mol% of Ru(OPiv)_2_(*p*-cymene) as the catalyst under optimized conditions in 1,4-dioxane and found that the reaction proceeded with 100% conversion and formation of the products **2a**/**3a** in a molar ratio of 23/77, which is similar to the result obtained with the catalyst system [RuCl_2_(*p*-cymene)]_2_/KOPiv (see Table 1). The free *p*-cymene ligand was also detected by ^1^H NMR spectroscopy in the crude reaction mixture. These results indicate that [RuCl_2_(*p*-cymene)]_2_ likely forms the active catalytic species Ru(OPiv)_2_L_3_ (L = solvent or **1a**) in situ. While the reaction of 2-bromo-3-methylpyridine (**1a**) with 5 mol% of [RuCl_2_(*p*-cymene)]_2_ in the presence of 1.25 equivalents of Na_2_CO_3_ resulted in only a 52% conversion at a molar ratio of **2a**/**3a** = 2/7, the addition of a catalytic amount of pyridyl pivalate **6** (20 mol%) to the reaction mixture led to the quantitative formation of pyridone **3a** (Scheme 5a). The abovementioned experiments suggest that pivalate is beneficial for successful conversion of **1a** and can be regenerated during the catalytic cycle. We suspected that the pyridone oxygen atom originated from the carbonate base; therefore, the reaction was studied with ^18^O-labeled Na_2_CO_3_ (Scheme 5b). The reaction was carried out in 1,4-dioxane at 150 °C for 72 h with 0.176 mmol of 2-bromopyridine **1a**, resulting in a 40% conversion to products **2a′** and **3a′**, which showed ~23% ^18^O-incorporation as evidenced by HRMS analysis of the crude reaction mixture as well as the isolated products **2a′** and **3a′**. Since 3-methylpyridin-2(1*H*)-one (**7**) could not be detected in the reaction mixture, we assumed that the pyridone **7** formed in situ reacted immediately to give *N*-pyridylpyridone **2**. To prove this, the reaction of pyridone **7** with 2-bromo-3-methylpyridine (**1a**) was carried out under the optimized reaction conditions in 1,4-dioxane. Pleasingly, the pyridone **3a** was formed quantitatively, indicating that the pyridone **7** can be *N*-arylated with **1a** to give first **2a** and then **3a** via C–H functionalization under the given reaction conditions (Scheme 5c). In contrast, when the reaction between pyridone **7** and bromopyridine **1a** was carried out without [RuCl_2_(*p*-cymene)]_2_, only the starting materials could be detected by ^1^H NMR analysis of the crude reaction mixture.

**Scheme 6:**
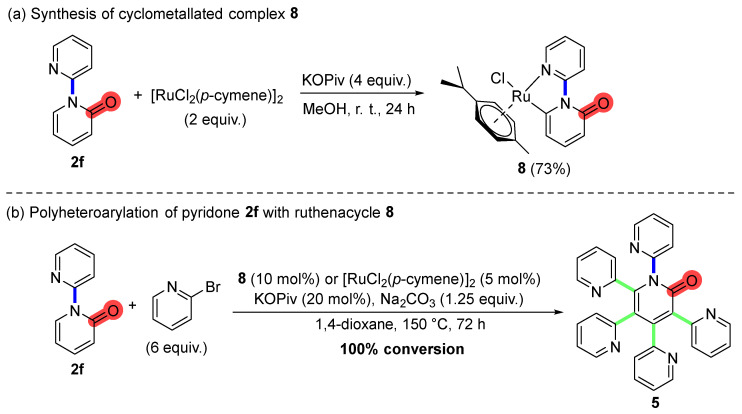
Synthesis of and catalysis with cyclometallated complex **8**.

Furthermore, we investigated the possibility of the formation of a ruthenacycle as an intermediate in the plausible C–H heteroarylation of pyridylpyridones **2**. Pleasingly, we were able to isolate metallacycle **8** in a 73% yield from the reaction of **2f** with two equivalents of [RuCl_2_(*p*-cymene)]_2_ by applying the protocol reported by Dixneuf et al. for the synthesis of five-membered ruthenacycles from arenes with some N-containing functionalities (Scheme 6a) [48,49]. Ruthenacycle **8** successfully catalyzed the polyheteroarylation of pyridone **2f** with 2-bromopyridine and provided an identical reaction outcome as the precatalyst [RuCl_2_(*p*-cymene)]_2_. NMR analysis showed that the desired product **5** arising from four consecutive C–H bond arylations of pyridone **2f** was formed with quantitative conversion in both cases (Scheme 6b).

**Scheme 7:**
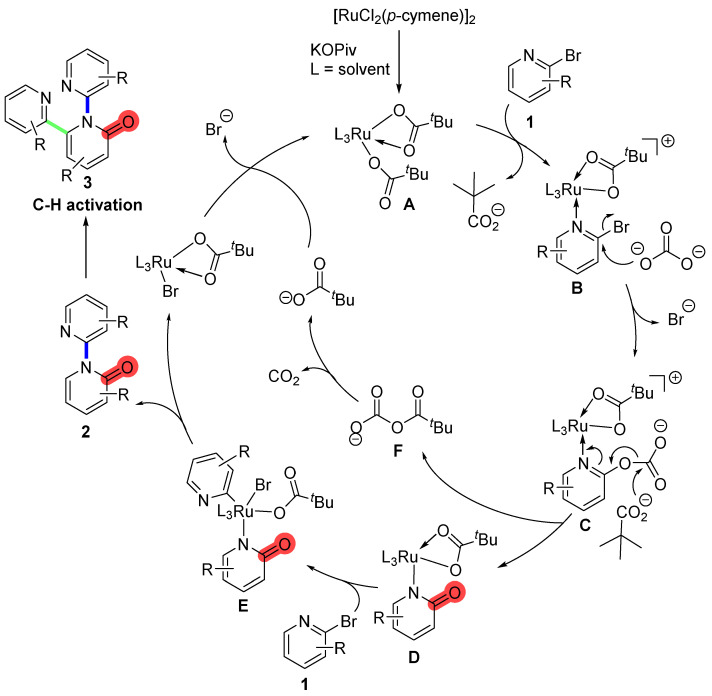
Plausible mechanism for 2-pyridone formation.

Based on the above experiments, a plausible pathway for the conversion of 2-bromopyridines **1** to 2-pyridones **2** was proposed (Scheme 7). First, [RuCl_2_(*p*-cymene)]_2_ together with the solvent and KOPiv most likely forms a *p*-cymene-free catalytic species **A**. The Ru(II) species **A** then coordinates to 2-bromopyridine, whereupon an intermediate **C** is formed by nucleophilic aromatic substitution of the bromine atom with carbonate. Then pivalate adds to the carbon atom of the carbonate in **C**, which leads to the formation of pyridone **D** and the elimination of the intermediate **F**. The intermediate **F** spontaneously decarboxylates to regenerate the pivalate anion for the next catalytic cycle. Complex **D** undergoes oxidative addition with 2-bromopyridine to form the Ru(IV) intermediate **E**, followed by reductive elimination of *N*-pyridylpyridone **2** and regeneration of the ruthenium catalyst. In the next step, the C–H bond functionalization of pyridone **2** directed by a 2-pyridyl group leads to the C-6-heteroarylated product **3**.

## 3. Materials and Methods

### 3.1. General Information

All reagents were commercial-grade and used without further purification. Reactions were monitored by analytical thin-layer chromatography (TLC) on Fluka silica gel TLC. Column chromatography was performed on 230–400 mesh silica gel. Merck silica gel 60 (PF254 containing gypsum (Merck Group, Darmstadt, Germany) was used to prepare chromatotron plates. Radial chromatography was performed with a Harrison Research chromatotron, model 7924 T (Harrison Research, Palo Alto, CA, USA). Melting points were determined on a Kofler micro hot stage instrument and with an SRS OptiMelt MPA100-Automated Melting Point System (Stanford Research System, Sunnyvale, CA, USA) and were uncorrected. The NMR spectroscopy data were recorded at 296 K with a Bruker Avance III at 500 MHz for ^1^H NMR and 125 MHz for ^13^C NMR (Bruker, Billerica, MA, USA). All NMR data were recorded in a CDCl_3_ and are given in ppm (δ). Chemical shifts for ^1^H NMR were referenced to TMS as an internal standard. The ^13^C NMR data were referenced against the central line of the CHCl_3_ triplet at δ 77.16 ppm. The coupling constants are given in Hertz (Hz). For the multiplicity signification, the standard abbreviations were used: s (singlet), d (doublet), t (triplet), q (quartet), sept (septet), m (multiplet), and br (broad). IR spectra were obtained with a Bruker ALPHA FT-IR spectrophotometer and reported in reciprocal centimeters (cm^−1^). High-resolution mass spectra were recorded with an Agilent 6224 Accurate Mass TOF LC/MS instrument (Agilent Technologies, Santa Clara, CA, USA). Elemental analyses (C, H, N) were performed with a Perkin-Elmer 2400 Series II CHNS/O Analyzer (PerkinElmer, Inc., Waltham, MA, USA).

### 3.2. General Procedure for Synthesis of Pyridones **2**–**5**

A high-pressure tube was loaded with substituted 2-bromopyridine (1 mmol), [RuCl_2_(*p*-cymene)]_2_ (30 mg, 0.05 mmol), potassium pivalate (KOPiv) (28 mg, 0.20 mmol), and Na_2_CO_3_ (123 mg, 1.25 mmol). The mixture was suspended in 1 mL of toluene, bubbled with argon for 3 min, and heated at 150 °C for 72 h. The reaction mixture was then cooled to room temperature and diluted with 10 mL DCM and 10 mL H_2_O. The product was extracted with DCM (2 × 10 mL). The combined organic phases were dried over anhydrous Na_2_SO_4_, filtered, and evaporated in vacuo. The crude product was further purified by radial chromatography on silica gel using a mixture of DCM and MeOH.

The following compounds were prepared using this general procedure:

*5,5′-Bis(trifluoromethyl)-2H-[1,2′-bipyridin]-2-one (***2b***)*. Prepared from 2-bromo-5-(trifluoromethyl)pyridine (226 mg, 1 mmol). Radial chromatography (DCM/MeOH: 100/1) yielded **2b** (90 mg, 0.285 mmol 57%) as a white solid. Mp. 145–147 °C. ^1^H NMR (500 MHz, CDCl_3_): δ 8.90–8.82 (m, 1H), 8.47–8.46 (m, 1H), 8.23 (d, *J* = 8.6 Hz, 1H), 8.12 (dd, *J* = 8.6, 2.4 Hz, 1H), 7.54 (dd, *J* = 9.7, 2.7 Hz, 1H), 6.75 (d, *J* = 9.6 Hz, 1H). ^13^C NMR (125 MHz, CDCl_3_): δ 161.3, 153.2, 146.3 (q, *J* = 4.1 Hz), 136.0 (q, *J* = 2.3 Hz), 135.7 (q, *J* = 3.4 Hz), 135.4 (q, *J* = 5.6 Hz), 126.8 (q, *J* = 79.5 Hz), 123.3 (q, *J* = 270.2 Hz), 123.2, 123.1 (q, *J* = 272.5 Hz), 121.0, 111.1 (q, *J* = 35.2 Hz). HRMS (ESI) *m*/*z*: [M + H]^+^ calcd for C_12_H_7_F_6_N_2_O, 309.0457; found, 309.0461. FT-IR (ATR): ν_max_/cm^−1^ 3068, 1686, 1637, 1600, 1550, 1323, 1276, 1231, 1116, 1081, 1061, 845, 721, 642, 626. Analytical data agree with the literature data [50].

*2-Oxo-2H-[1,2′-bipyridine]-3,3′-dicarbonitrile (***2d***)*. Prepared from 2-bromonicotinonitrile (183 mg, 1 mmol). Radial chromatography (DCM/MeOH: 100/1 to 25/1) yielded **2d** (50 mg, 0.21 mmol, 42%) as a white solid. Mp. 200–202 °C (dec.). ^1^H NMR (500 MHz, CDCl_3_): δ 8.83 (dd, *J* = 4.9, 1.8 Hz, 1H), 8.22 (dd, *J* = 7.8, 1.8 Hz, 1H), 7.98 (dd, *J* = 7.0, 2.1 Hz, 1H), 7.77 (dd, *J* = 7.0, 2.1 Hz, 1H), 7.62 (dd, *J* = 7.8, 4.9 Hz, 1H), 6.48 (t, *J* = 7.0 Hz, 1H). ^13^C NMR (125 MHz, CDCl_3_): δ 158.2, 153.0, 152.1, 149.0, 142.6, 141.0, 124.7, 114.7, 113.9, 109.3, 107.6, 106.3. HRMS (ESI) *m*/*z*: [M + H]^+^ calcd for C_12_H_7_N_4_O, 223.0614; found, 223.0613. FT-IR (ATR): ν_max_/cm^−1^ 3123, 3061, 2239, 226, 1656, 1600, 1586, 1540, 0430, 1360, 1276, 1076, 759. Analytical data agree with the literature data [51].

*3,3″,5′-Trimethyl-6′H-[2,1′:2′,2″-terpyridin]-6′-one (***3a***)*. Prepared from 2-bromo-3-methylpyridine (123 µL, 1 mmol). Radial chromatography (DCM/MeOH: 100/1 to 50/1) yielded **3a** (80 mg, 0.277 mmol, 83%) as a pale-brown solid. Mp. 145–150 °C. ^1^H NMR (500 MHz, CDCl_3_): δ 8.08 (dd, *J* = 4.7, 1.6 Hz, 1H), 8.03 (dd, *J* = 4.8, 1.7 Hz, 1H), 7.42 (dd, *J* = 7.6, 1.7 Hz, 1H), 7.40–7.30 (m, 2H), 6.97 (dd, *J* = 7.6, 4.8 Hz, 1H), 6.93 (dd, *J* = 7.7, 4.7 Hz, 1H), 6.14 (d, *J* = 6.8 Hz, 1H), 2.37 (s, 3H), 2.31 (s, 3H), 2.22 (s, 3H). ^13^C NMR (125 MHz, CDCl_3_): δ 162.8, 152.8, 151.6, 146.0, 145.9, 144.1, 138.8, 137.8, 136.8, 133.3, 133.1, 129.9, 123.7, 123.2, 106.6, 19.4, 17.7, 17.2. HRMS (ESI) *m*/*z*: [M + H]^+^ calcd for C_18_H_18_N_3_O, 292.1444; found, 292.1446. FT-IR (ATR): ν_max_/cm^−1^ 3051, 2974, 2922, 1651, 1601, 1574, 1418, 1267, 1116, 807, 791, 796.

*4,4′,4″-Tris(trifluoromethyl)-6′H-[2,1′:2′,2″-terpyridin]-6′-one (***3c***).* Prepared from 2-bromo-4-(trifluoromethyl)pyridine (123 µL, 1 mmol). Radial chromatography (DCM/MeOH: 100/1) yielded **3c** (57 mg, 0.127 mmol, 38%) as a red-brown oil. ^1^H NMR (500 MHz, CDCl_3_): δ 8.38 (d, *J* = 5.0 Hz, 1H), 8.32 (d, *J* = 5.1 Hz, 1H), 7.93 (s, 1H), 7.40 (d, *J* = 4.9 Hz, 1H), 7.37 (d, *J* = 4.7 Hz, 2H), 7.06 (s, 1H), 6.64 (s, 1H). ^13^C NMR (125 MHz, CDCl_3_): δ 161.7, 153.9, 152.0, 150.0, 149.5, 147.5, 141.5 (q, *J* = 34.3 Hz), 139.9 (q, *J* = 34.8 Hz), 139.6 (q, *J* = 34.7 Hz), 122.3 (q, *J* = 273.4 Hz), 122.2 (q, *J* = 273.6 Hz), 121.9 (q, *J* = 274.4 Hz), 121.8 (q, *J* = 3.7 Hz), 120.2 (q, *J* = 4.3 Hz), 119.6 (q, *J* = 3.6 Hz), 119.2 (q, *J* = 3.3 Hz), 119.0 (q, *J* = 3.4 Hz), 104.4 (q, *J* = 2.7 Hz). HRMS (ESI) *m*/*z*: [M + H]^+^ calcd for C_9_H_9_F_9_N_3_O, 454.0596; found, 454.0591. FT-IR (ATR): ν_max_/cm^−1^ 3079, 2017, 1685, 1405, 1327, 1135, 902, 838, 666.

4,4′,4″-Trimethyl-6′H-[2,1′:2′,2″-terpyridin]-6′-one (**3e**) and 4,4′,4″-trimethyl-3′-(4-methylpyridin-2-yl)-6′H-[2,1′:2′,2″-terpyridin]-6′-one (**4e**). Prepared from 2-bromo-4-methylpyridine (111 µL, 1 mmol). Radial chromatography (DCM/MeOH: 100/1 to 25/1) yielded **3e** (15 mg, 0.075 mmol, 15%) as a pale-brown oil and **4e** (30 mg, 0.107 mmol, 32%) as a brown oil. Data for **3e**: ^1^H NMR (500 MHz, CDCl_3_): δ 8.12 (d, J = 5.0 Hz, 1H), 8.06 (d, J = 5.1 Hz, 1H), 7.38 (s, 1H), 7.22 (s, 1H), 6.93 (d, J = 5.1 Hz, 1H), 6.89 (d, J = 5.0 Hz, 1H), 6.53 (s, 1H), 6.29 (s, 1H), 2.36 (s, 3H), 2.28 (s, 3H), 2.27 (s, 3H). ^13^C NMR (125 MHz, CDCl_3_): δ 163.2, 153.7, 152.0, 151.3, 148.8, 148.4, 148.0, 147.8, 146.4, 125.9, 125.1, 124.1, 123.7, 119.8, 111.4, 27.3, 21.6, 21.1. HRMS (ESI) m/z: [M + H]^+^ calcd for C_18_H_18_N_3_O, 292.1444; found, 292.1442. FT-IR (ATR): ν_max_/cm^−1^ 3049, 2921, 1659, 1611, 1597, 1544, 1429, 1276, 994, 822, 729. Data for **4e**: ^1^H NMR (500 MHz, CDCl_3_): δ 8.40 (d, J = 5.0 Hz, 1H), 8.07 (d, J = 5.1 Hz, 1H), 7.99 (d, J = 5.0 Hz, 1H), 7.28–7.22 (m, 1H), 6.92–6.84 (m, 3H), 6.77 (s, 1H), 6.67 (d, J = 1.2 Hz, 1H), 6.64 (dd, J = 5.1, 1.2 Hz, 1H), 2.30 (s, 3H), 2.10 (s, 3H), 2.08 (s, 3H), 2.02 (s, 3H).^13^C{1H} NMR (126 MHz, CDCl_3_): δ 162.3, 155.4, 152.4, 151.8, 151.5, 148.8, 148.7, 148.2, 147.7, 147.2, 146.6, 144.6, 127.9, 127.4, 125.8, 124.1, 123.1, 122.8, 121.7, 120.5, 20.93, 20.90, 20.77, 20.76. HRMS (ESI) m/z: [M + H]^+^ calcd for C_22_H_21_N_4_O, 383.1866; found, 383.1859. FT-IR (ATR): ν_max_/cm^−1^ 3049, 2921, 1657, 1599, 1556, 1527, 1404, 994, 828, 730.

*3′,4′,5′-Tri(pyridin-2-yl)-6′H-[2,1′:2′,2″-terpyridin]-6′-one (***5***)*. Prepared from 2-bromopyridine (90 µL, 1 mmol). Radial chromatography (DCM/MeOH: 50/1 to 10/1) yielded **5** (51 mg, 0.107 mmol, 64%) as a brown solid. Mp. > 300 °C (dec.). ^1^H NMR (500 MHz, CDCl_3_): δ 8.33 (dt, *J* = 4.9, 1.4 Hz, 1H), 8.22 (dd, *J* = 5.0, 1.8 Hz, 1H), 8.20–8.14 (m, 2H), 8.16–8.09 (m, 1H), 7.67 (td, *J* = 7.7, 1.9 Hz, 1H), 7.56 (d, *J* = 8.0 Hz, 1H), 7.54–7.52 (m, 2H), 7.30–7.26 (m, 1H), 7.26–7.22 (m, 1H), 7.19 (td, *J* = 7.7, 1.8 Hz, 1H), 7.07 (d, *J* = 7.6 Hz, 2H), 7.03–6.97 (m, 2H), 6.92 (d, *J* = 7.9 Hz, 1H), 6.84 (dd, *J* = 7.5, 4.9 Hz, 2H), 6.78 (ddd, *J* = 7.6, 5.0, 1.2 Hz, 1H). ^13^C NMR (125 MHz, CDCl_3_): δ 161.9, 156.4, 155.5, 154.4, 152.8, 152.0, 151.0, 148.8, 148.7, 148.5, 148.4, 148.3, 145.5, 137.2, 135.3, 135.2, 135.0, 134.9, 131.1, 127.3, 127.1, 126.8, 125.7, 125.6, 123.0, 122.2, 121.8, 121.4, 121.1, 121.0. HRMS (ESI) *m*/*z*: [M + 2H]^2+^ calcd for C_30_H_22_N_6_O, 241.092; found, 241.0919. FT-IR (ATR): ν_max_/cm^−1^ 3050, 1650, 1582, 1564, 1527, 1463, 1430, 992, 762, 745. Elemental analysis calcd (%) for C_30_H_20_N_6_O x H_2_O: C 72.28, H 4.45, N 16.86; found: C 72.56, H 4.05, N 16.84.

*Synthesis of 3,3′-Dimethyl-2H-[1,2′-bipyridin]-2-one (***2a***)*. A high-pressure tube was loaded with 2-bromo-3-methylpyridine (123 µL, 1 mmol), [RuCl_2_(*p*-cymene)]_2_ (30 mg, 0.05 mmol), potassium pivalate (KOPiv) (28 mg, 0.20 mmol), and Na_2_CO_3_ (123 mg, 1.25 mmol). The mixture was suspended in 1 mL of 1,4-dioxane, bubbled with Ar for 3 min and heated at 150 °C for 72 h. The reaction mixture was then cooled to room temperature and diluted with 10 mL DCM and 10 mL H_2_O. The product was extracted with DCM (2 × 10 mL). The combined organic phases were dried over anh. Na_2_SO_4_, filtered and evaporated in vacuo. The crude product (a mixture of **2a**:**3a** 20/80) was further purified by radial chromatography on silica gel using a mixture of DCM and MeOH (100/1 to 25/1) to obtain pyridone **2a** (12 mg, 0.06 mmol, 12%) as pale-yellow oil and pyridone **3a** (59 mg, 0.207 mmol, 62%) as pale brown solid. Data for **2a**: ^1^H NMR (500 MHz, CDCl_3_): δ 8.42 (dd, *J* = 4.8, 1.7 Hz, 1H), 7.68 (dd, *J* = 7.6, 1.8 Hz, 1H), 7.33 − 7.27 (m, 2H), 7.22 (dd, *J* = 6.9, 2.0 Hz, 1H), 6.20 (t, *J* = 6.8 Hz, 1H), 2.21 (s, 3H), 2.19 (s, 3H). ^13^C NMR (125 MHz, CDCl_3_): δ 162.0, 152.6, 147.0, 139.8, 137.4, 134.1, 131.0, 130.6, 124.3, 105.8, 17.4, 17.1. HRMS (ESI) *m*/*z*: [M + H]^+^ calcd for C_12_H_13_N_2_O, 201.1022; found, 201.1023. FT-IR (ATR): ν_max_/cm^−1^ 2923, 1652, 1603, 1553, 1451, 1419, 1274, 759. Analytical data agree with the literature data [52].

*Synthesis of 3-Methylpyridin-2-yl pivalate (***6***)*. Compound **6** was prepared by following the literature procedure for a similar compound [53]. In a high-pressure tube, pyridone **7** (109 mg, 1 mmol) and pivaloyl chloride (134 µL, 1.1 mmol) were mixed in dry toluene (1 mL). After DMAP × HCl (7.9 mg, 0.05 mmol, 5 mol%) was added and the reaction mixture was stirred at 110 °C for 30 min under an Ar atmosphere. After that, the mixture was allowed to cool to room temperature, and then hexane (5 mL) was added. DMAP·HCl was precipitated and removed by filtration. The filtrate was extracted with saturated Na_2_HCO_3_ solution (2 × 5 mL). The organic phase was dried over anhydrous Na_2_SO_2_, filtrated, and evaporated in vacuo to obtain the pure product **6** (144 mg, 0.75 mmol, 75%) as a colorless oil. ^1^H NMR (500 MHz, CDCl_3_): δ 8.24 (dd, *J* = 5.0, 1.9 Hz, 1H), 7.59 (dd, *J* = 7.4, 1.7 Hz, 1H), 7.14 (dd, *J* = 7.4, 4.8 Hz, 1H), 2.19 (s, 3H), 1.41 (s, 9H). ^13^C NMR (125 MHz, CDCl_3_): δ 176.4, 157.2, 146.1, 140.6, 125.6, 122.4, 27.2, 26.6, 16.0. HRMS (ESI) *m*/*z*: [M + H]^+^ calcd for C_11_H_16_NO_2_, 194.1181; found, 194.1177. FT-IR (ATR): ν_max_/cm^−1^ 2908, 1666, 1635, 1605, 1475, 126, 971, 915, 772, 737.

*Synthesis of 2H-[1,2′-bipyridin]-2-one (***2f***)*. An oven-dried high-pressure tube was charged with 2-hydroxypyridine (95 mg, 1 mmol), CuI (38 mg, 0.2 mmol, 20 mol%), and K_2_CO_3_ (2 mmol, 2 equiv.). The flask was purged with nitrogen. A solution of 2-bromopyridine (90 µL, 1 mmol) in toluene (1 mL) and TMEDA (42 µL, 0.2 mmol, 20 mol%) were added to the reaction mixture, which was stirred and heated 110 °C for 16 h. The resulting mixture was cooled to room temperature, diluted with dichloromethane, and filtered. The filtrate was washed with water. The organic phase was collected, dried over anhydrous Na_2_SO_4_, filtered, and concentrated in vacuo. The crude product was purified by column chromatography to obtain product **2f** (86 mg, 0.50 mmol, 50%) as a white solid. Mp. 50–54 °C. ^1^H NMR (500 MHz, CDCl_3_): δ 8.57 (dd, *J* = 5.0, 2.7 Hz, 1H), 7.93 (d, *J* = 8.1 Hz, 1H), 7.88 (dd, *J* = 7.2, 2.1 Hz, 1H), 7.84 (td, *J* = 7.8, 1.9 Hz, 1H), 7.40 (ddd, *J* = 9.0, 6.5, 2.1 Hz, 1H), 7.33 (ddd, *J* = 7.4, 4.9, 1.1 Hz, 1H), 6.65 (d, *J* = 9.2 Hz, 1H), 6.30 (td, *J* = 6.8, 1.3 Hz, 1H). HRMS (ESI) *m*/*z*: [M + H]^+^ calcd for C_10_H_9_N_2_O, 173.0715; found, 173.0711. FT-IR (ATR): ν_max_/cm^−1^ 3058, 2991, 1673, 1612, 1540, 1175, 1130, 999, 785. Analytical data agree with the literature data [47].

*Synthesis of ruthenacycle* **8**. Ruthenacycle was prepared by following slightly modified literature procedure [48]. A mixture of pyridone **2f** (8.6 mg, 0.05 mmol), [RuCl_2_(*p*-cymene)]_2_ (61.4 mg, 0.1 mmol), and KOPiv (28 mg, 0.2 mmol) was dissolved in MeOH (1 mL) and then mixed at room temperature for 24 h. A solvent was then evaporated in vacuo and the crude product was further purified by radial chromatography on silica gel using ethyl acetate to give ruthenacycle **8** (32 mg, 0.037 mmol, 73%) as an orange semisolid. ^1^H NMR (500 MHz, CDCl_3_): δ 9.38 (dd, *J* = 8.6, 1.5 Hz, 1H), 9.10 (dd, *J* = 5.7, 1.8 Hz, 1H), 7.81 (ddd, *J* = 9.0, 7.3, 1.8 Hz, 1H), 7.16 (ddd, *J* = 7.2, 5.8, 1.3 Hz, 1H), 7.09 (dd, *J* = 9.0, 6.8 Hz, 1H), 6.94 (dd, *J* = 6.9, 1.4 Hz, 1H), 6.14 (dd, *J* = 8.9, 1.3 Hz, 1H), 5.68 − 5.55 (m, 2H), 5.28 (dd, *J* = 6.1, 1.3 Hz, 1H), 5.08 (dd, *J* = 6.1, 1.3 Hz, 1H), 2.52 (sept, *J* = 6.9 Hz, 1H), 2.12 (s, 3H), 1.05 (d, *J* = 6.9 Hz, 3H), 0.96 (d, *J* = 6.9 Hz, 3H). ^13^C NMR (125 MHz, CDCl_3_): δ 186.8, 166.6, 158.8, 153.9, 139.2, 138.5, 121.7, 119.0, 118.0, 113.7, 103.8, 103.2, 92.0, 91.3, 86.0, 84.4, 30.8, 22.6, 22.1, 18.8. HRMS (ESI) *m*/*z*: [M + H]^+^ calcd for C_20_H_22_ClN_2_ORu, 437.0491; found, 437.0495. FT-IR (ATR): ν_max_/cm^−1^ 2958, 1634, 1498, 1464, 1434, 1277, 1186, 1135, 1000, 785.

*Reaction with ^18^O-labeled Na_2_CO_3_*. A high-pressure tube was loaded with substituted 2-bromopyridine **1a** (20 µL, 0.176 mmol), [RuCl_2_(*p*-cymene)]_2_ (5 mg, 0.0088 mmol), KOPiv (5 mg, 0.035 mmol), and Na_2_CO_3_ (25 mg, 0.22 mmol). The mixture was suspended in 200 µL of 1,4-dioxane, bubbled with Ar for 3 min, and heated at 150 °C for 72 h. The reaction mixture was then cooled to room temperature and diluted with 3 mL DCM and 3 mL H_2_O. The product was extracted with DCM (2 × 3 mL). The combined organic phases were dried over anh. Na_2_SO_4_, filtered and evaporated in vacuo. The crude product was further purified by radial chromatography on silica gel using a mixture of DCM and MeOH (100/1 to 25/1) to obtain pyridone **2a′** (3 mg, 0.015 mmol, 17%) and **3a′** (4 mg, 0.013 mmol, 23%). Data for **2a′**: HRMS (ESI) *m*/*z*: [M + H]^+^ calcd for C_12_H_13_N_2_^18^O, 203.1065; found, 203.1107. Data for **3a′**: HRMS (ESI) *m*/*z*: [M + H]^+^ calcd for C_18_H_18_N_3_^18^O, 294.1487; found, 294.1534. The ^1^H NMR data agree with those of pyridones **2a** and **3a**.

## 4. Conclusions

In summary, we have developed a novel synthetic route to polyheteroarylated 2-pyridones from various substituted 2-bromopyridines. As a result, 2-pyridones with different degrees of 2-pyridyl substitution and, in particular, pyridone with one (2-pyridyl) C–N bond and with 2-, 4- ,5-, and 6-(2-pyridyl) C–C bonds, can now be readily prepared. The small scope of the various 2-bromopyridine starting compounds indicates that electron-withdrawing groups have an advantage over electron-donating groups in pyridone formation. The preliminary mechanistic study suggests that the oxygen required for 2-pyridone formation is derived from carbonate. Although the yields of 2-pyridone products were only moderate, the catalytic method represents an interesting synthetic route to construct complex molecular scaffolds from simple precursors. The prepared 2-pyridone derivatives can be used as ligands in coordination with transition metals for supramolecular architectures or for the preparation of multimetallic catalytically active species.

## Figures and Tables

**Table 1:** Optimization study of pyridone **3a** formation ^1^.

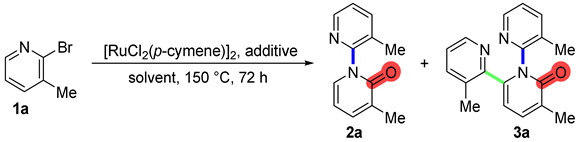				
Entry	[Ru] (mol%)	Additive (mol%)	Solvent	1a/2a/3a ^2^
1	(1.25)	KOPiv (20)/PPh_3_ (2.5)/Na_2_CO_3_ (125)	1,4-dioxane	25/40/35
2	(1)	KOPiv (4)	1,4-dioxane	95/5/0
3	(1)	Na_2_CO_3_ (125)	1,4-dioxane	85/7/8
4	(1)	KOPiv (4)/Na_2_CO_3_ (125)	1,4-dioxane	0/80/20
**5**	**(5)**	**KOPiv (20)/Na_2_CO_3_ (125)**	**1,4-dioxane**	**0/20 (12%) ^3^/80 (62%) ^3^**
6	(5)	KOPiv (20)/Na_2_CO_3_ (125)	NMP	15/20/65
7	(5)	KOPiv (20)/Na_2_CO_3_ (125)	2-MeTHF	0/50/50
8	(5)	KOPiv (20)/Na_2_CO_3_ (125)	DEC	0/25/75
**9**	**(5)**	**KOPiv (20)/Na_2_CO_3_ (125)**	**toluene**	**0/5/95 (83) ^3^**
10	(5)	KOPiv (20)/Na_2_CO_3_ (125)	H_2_O	100/0/0
11	(1)	KOPiv (4)/Na_2_CO_3_ (125)	1,4-dioxane/H_2_O (4:1)	75/20/5

^1^ Reagents and conditions: 2-bromo-3-methylpyridine (**1a**) (1 mmol), solvent (1 mL), 150 °C argon. ^2^ Molar ratio determined by ^1^H NMR analysis. ^3^ Isolated yield. [Ru] = [RuCl_2_(*p*-cymene)]_2_.

## Data Availability

The data underlying this study are available in the published article and its Supporting Information.

## Supplementary Materials

The following supporting information can be downloaded at https://www.mdpi.com/article/10.3390/molecules29184418/s1: Table of crystallographic details, and the ^1^H and ^13^C NMR spectra. CCDC 2345218 contains the supplementary crystallographic data for this paper. These data can be obtained free of charge via www.ccdc.cam.ac.uk/data_request/cif (accessed on 22 August 2024) or by emailing data_request@ccdc.cam.ac.uk or by contacting The Cambridge Crystallography Data Centre, 12 Unior Road, Cambridge CB2 1EZ, UK; fax: +44 1223 336033. References [54,55,56,57].
