# Supplementary material for: 2-Bromopyridines as Versatile Synthons for Heteroarylated 2-Pyridones via Ru(II)-Mediated Domino C–O/C–N/C–C Bond Formation Reactions
Source: Molecules. 2024 Sep 17;29(18):4418. doi: 10.3390/molecules29184418 (PMC11433726; doi:10.3390/molecules29184418)

2-Bromopyridines as Versatile Synthons for Heteroarylated 2-Pyridones *via* Ru(II)-mediated Domino C–O/C–N/C–C Bond Formation Reactions

Miha Drev, Helena Brodnik, Uroš Grošelj, Franc Perdih, Jurij Svete, Bogdan Štefane, and Franc Požgan*

Faculty of Chemistry and Chemical Technology, University of Ljubljana, Večna pot 113, SI-1000 Ljubljana, Slovenia

Table of Contents

Crystal structure ………………………………………………………………………..3

References …………………………………………………………………………..4

NMR spectra of synthesized products ………………………………………………5

Crystal Structure

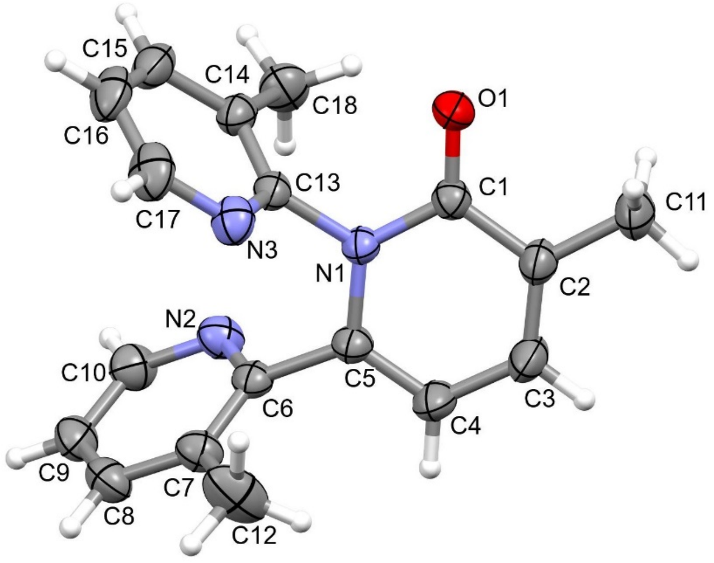

Figure S1. X-Ray crystal structure of **3a**

X-ray Crystallographic studies

Crystal data for **3a** were collected at room temperature on an Agilent Technologies SuperNova Dual diffractometer using monochromated Cu-*K*α radiation (λ = 1.54184 Å). The data were processed using CrysAlis Pro [1]. Structures was solved with the ShelXS [2] structure solution program and refined by the full-matrix least-squares procedure based on F^2 with ShelXL [3] implemented in Olex^2 program suit [4]. All non-hydrogen atoms were readily located and refined anisotropically unless otherwise noted. Hydrogen atoms were initially located in the difference Fourier maps and were subsequently included in the model at geometrically calculated positions and refined by using a riding model. Crystallographic data are listed in Table SI1.

Supplementary Materials: CCDC 2345218 contains the supplementary crystallographic data for this paper. These data can be obtained free of charge via www.ccdc.cam.ac.uk/data_request/cif or by emailing data_request@ccdc.cam.ac.uk or by contacting The Cambridge Crystallography Data Centre, 12 Unior Road, Cambridge CB2 1EZ, UK; fax: +44 1223 336033.

Table S1: Crystal data and structure refinement of **3a.**

	**3a**
CCDC number	2345218
Formula	$C_{18}H_{17}N_3O$
M_r	291.34

T (K)	293(2)
Crystal system	Triclinic
Space group	P–1
a (Å)	7.8360(7)
b (Å)	8.8245(8)
c (Å)	12.8873(12)
α (°)	77.761(7)
β (°)	81.239(8)
γ (°)	69.796(8)
Volume (Å^3)	814.26(14)
Z	2
D_c (g/cm^3)	1.188
μ (mm^{-1})	0.602
F(000)	308.0
Reflections collected	5980
Independent reflections (R_{int})	3096 (0.0424)
Data/restraints/parameters	3096/0/203
R, wR_2 [$I > 2\sigma(I)$]^a	0.0601, 0.1669
R, wR_2 (all data)^a	0.0778, 0.2000
GOF, S^b	1.045
Largest diff. peak/hole / e Å^{-3}	0.19/–0.21

^{a} $R = \sum||F_o| - |F_c||/\sum|F_o|$, $wR_2 = \{\sum[w(F_o^2 - F_c^2)^2]/\sum[w(F_o^2)^2]\}^{1/2}$. ^{b} $S = \{\sum[(F_o^2 - F_c^2)^2]/(n/p\}^{1/2}$, where n is the number of reflections and p is the total number of parameters refined.

[1] *CrysAlisPro*, version 1.171.36.28; Rigaku Oxford Diffraction: Yarnton, UK, 2013.

[2] Sheldrick, G.M. A short history of *SHELX. Acta Crystallogr.* **2008**, *A64*, 112–122.

[3] Sheldrick, G.M. Crystal structure refinement with SHELXL. *Acta Crystallogr.* **2015**, *C71*, 3–8.

[4] Dolomanov, O.V.; Bourhis, L.J.; Gildea, R.J.; Howard, J.A.K.; Puschmann, H. OLEX2: A complete structure solution, refinement and analysis program. *J. Appl. Crystallogr.* **2009**, 42, 339–341.

NMR spectra of synthesized products

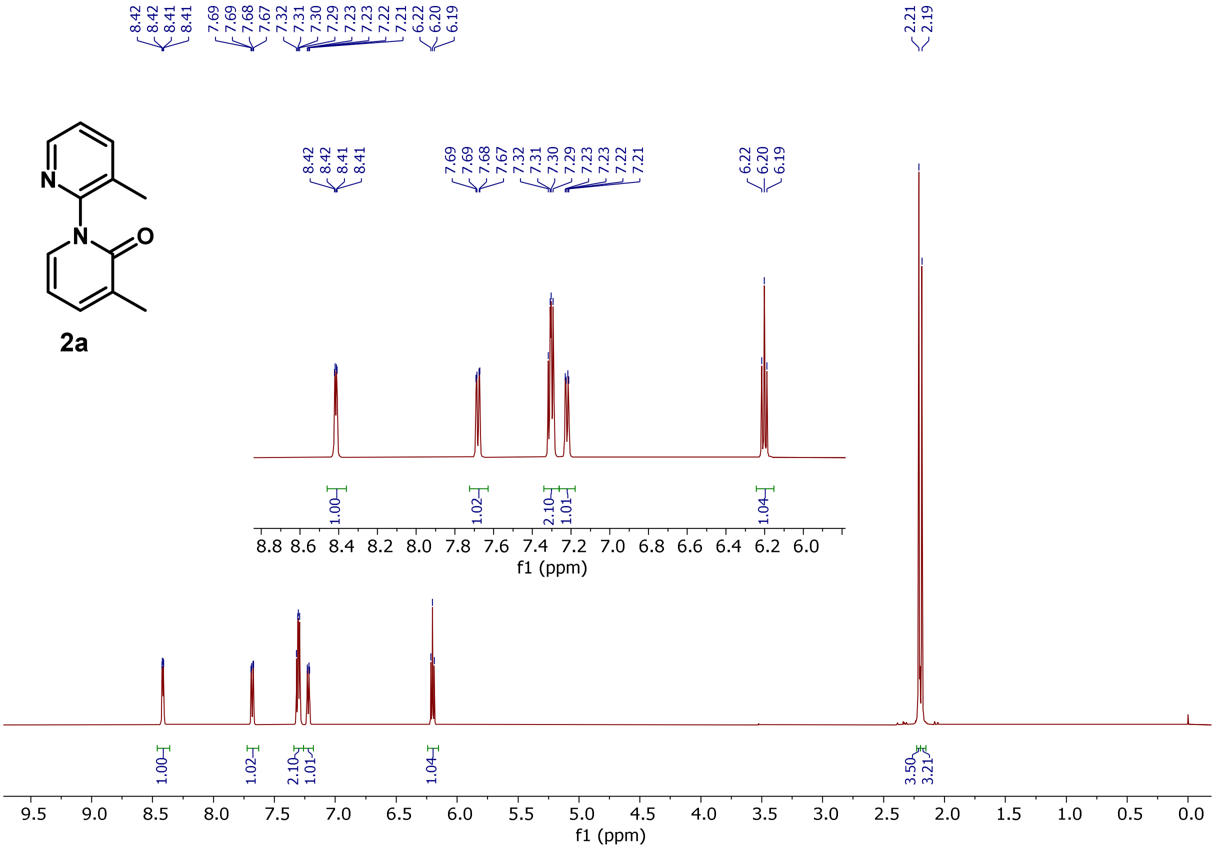

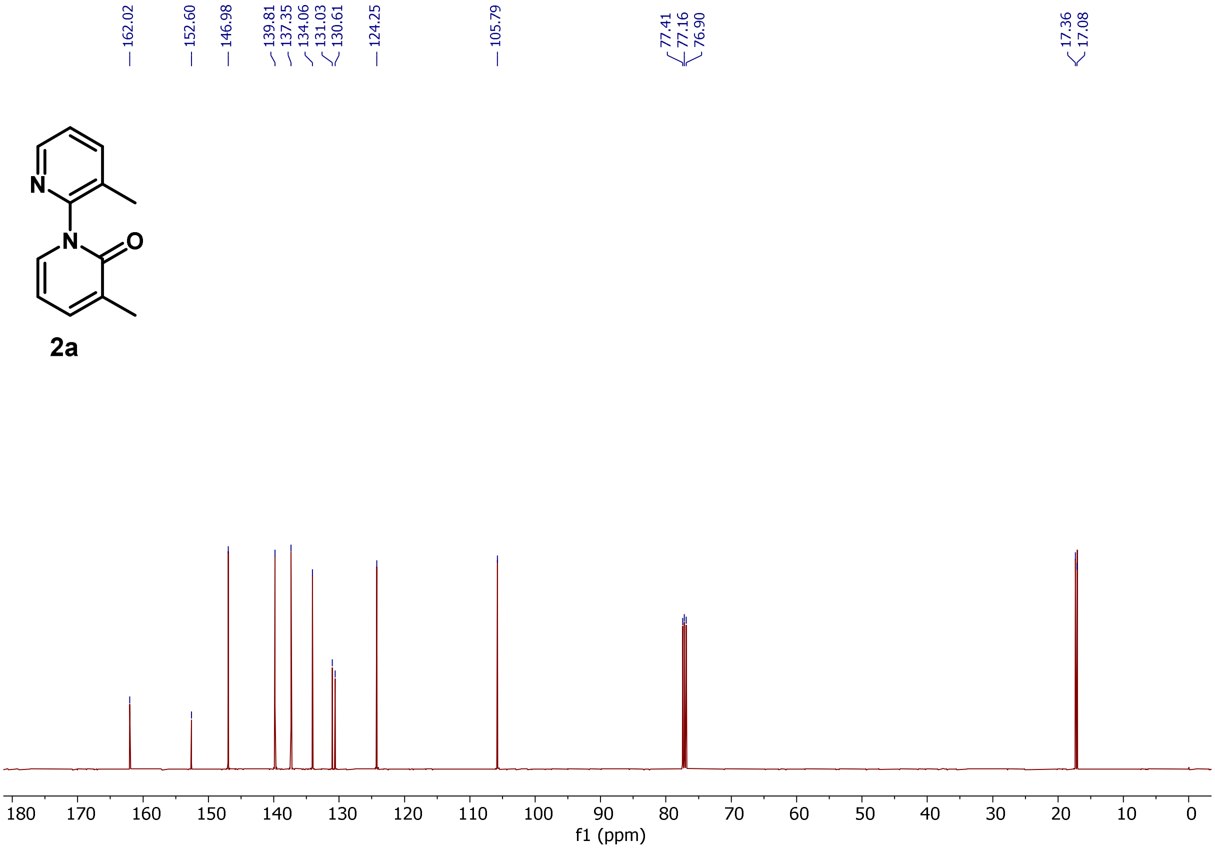

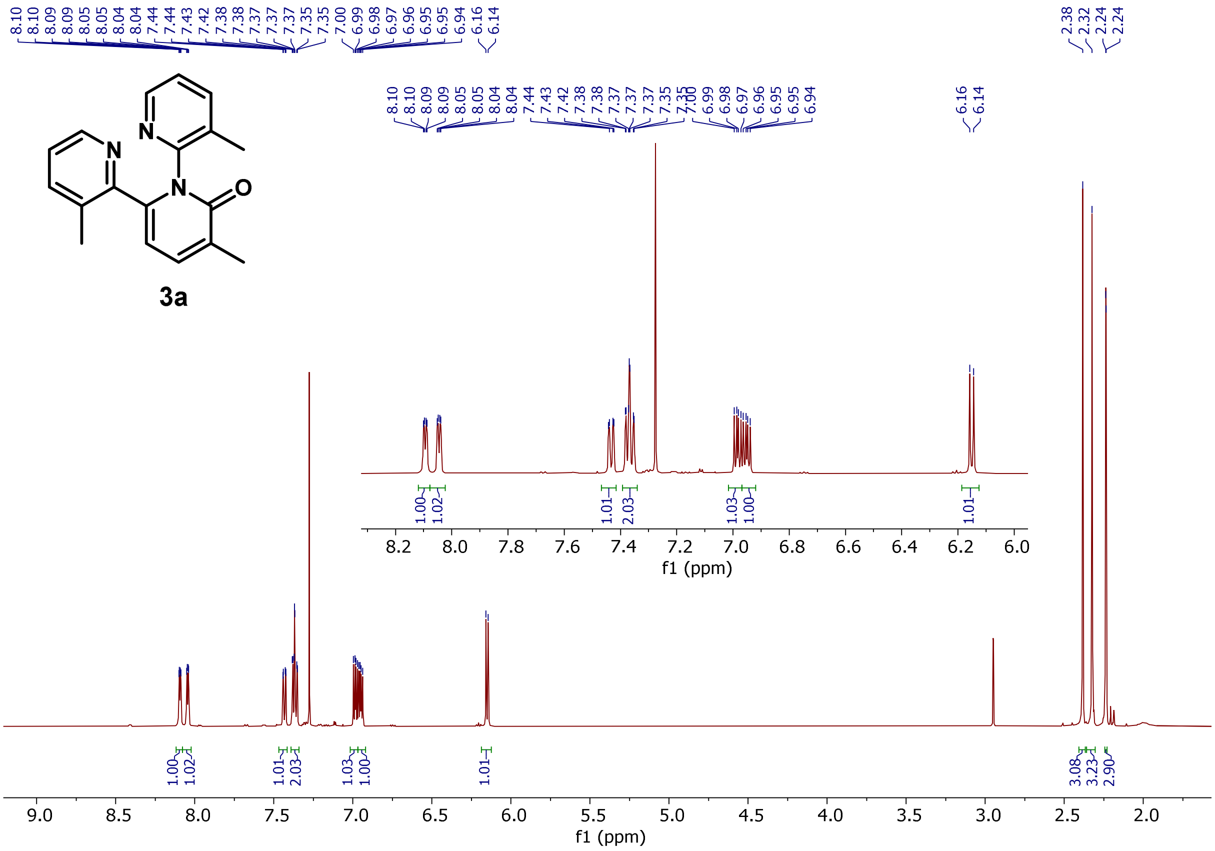
8.10 8.10 8.09 8.09 8.05 8.05 8.04 8.04 7.44 7.44 7.43 7.42 7.38 7.38 7.37 7.37 7.37 7.35 7.35 7.00 6.99 6.98 6.97 6.96 6.95 6.95 6.94 6.16 6.14
2.38 2.32 2.24 2.24
3a
1.00 1.02 1.01 2.03 1.03 1.00 1.01 3.08 3.23 2.90
f1 (ppm)

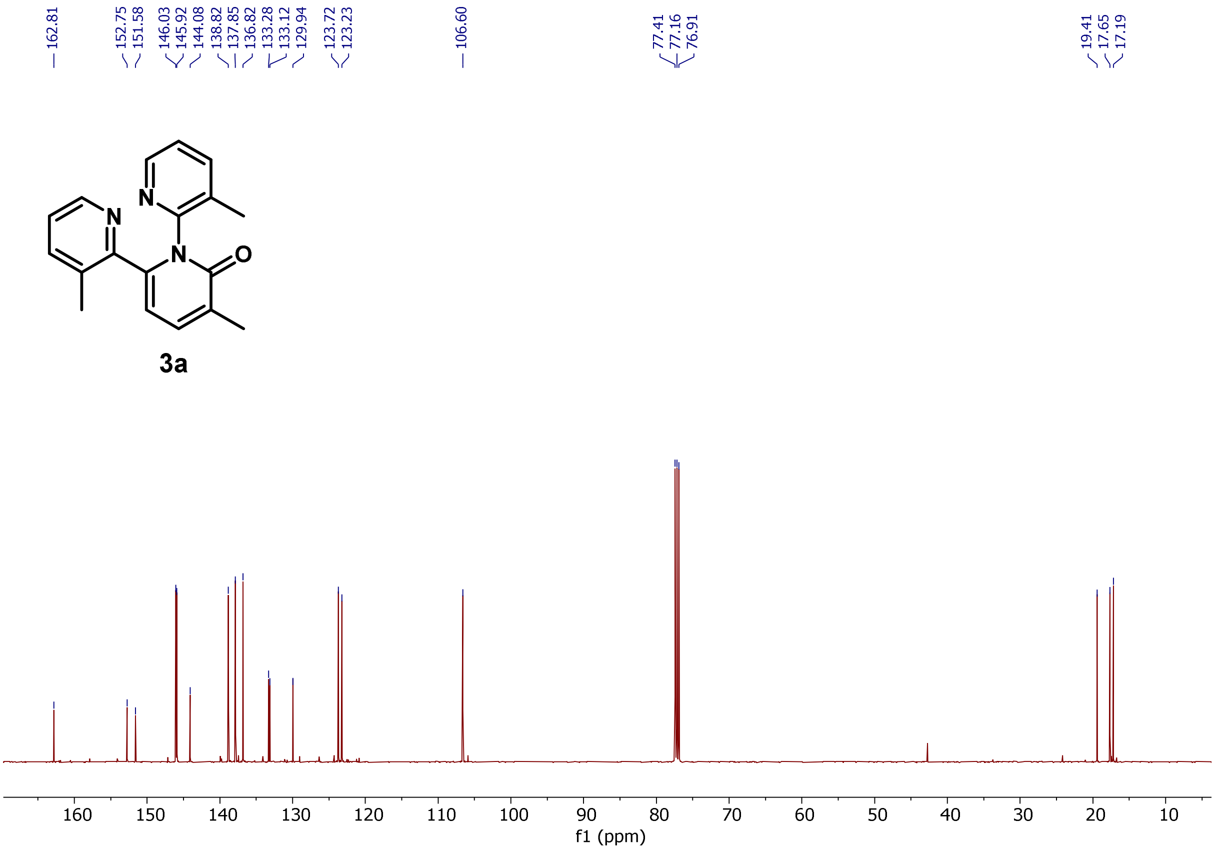
162.81 152.75 151.58 146.03 145.92 144.08 138.82 137.85 136.82 133.28 133.12 129.94 123.72 123.23 106.60 77.41 77.16 76.91 19.41 17.65 17.19
3a
f1 (ppm)

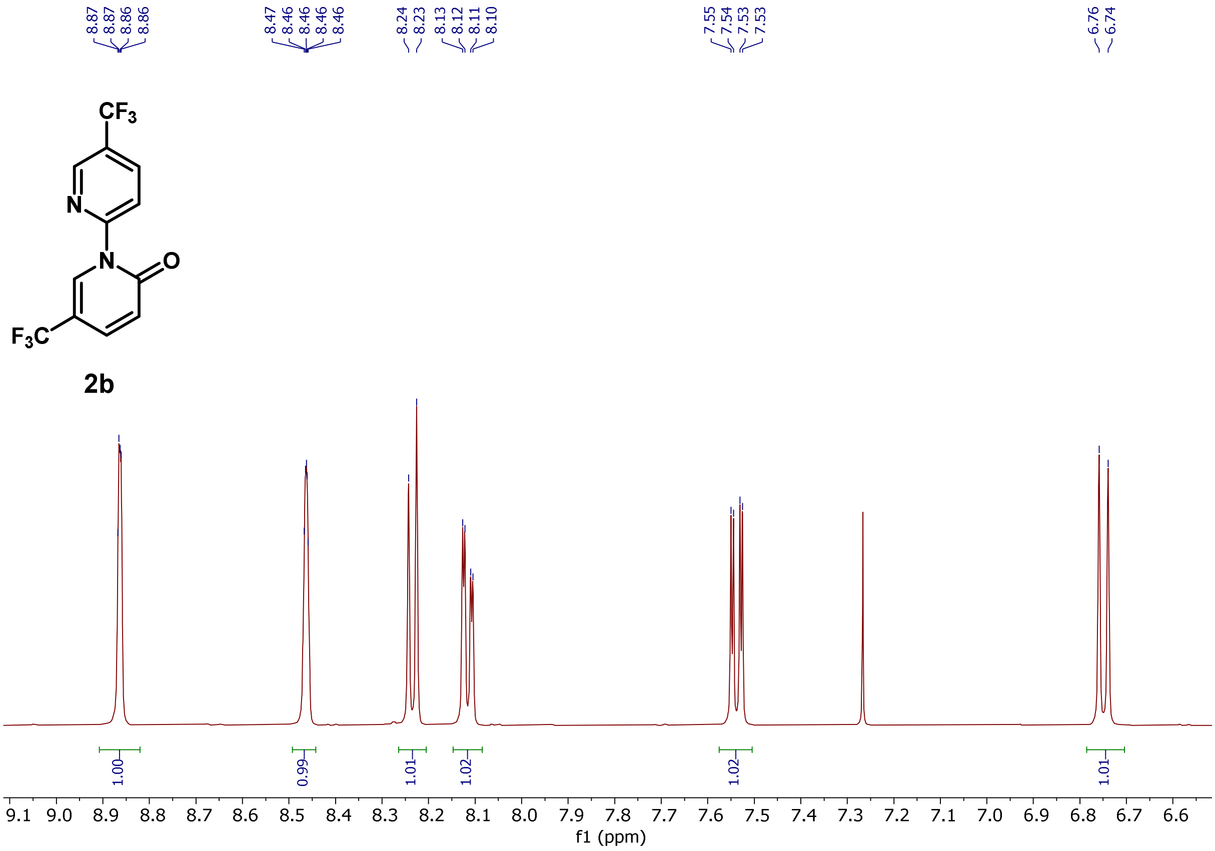
8.87
8.87
8.86
8.86
8.47
8.46
8.46
8.46
8.46
8.24
8.23
8.13
8.12
8.11
8.10
7.55
7.54
7.53
7.53
6.76
6.74
CF3
N
N
O
F3C
2b
1.00
0.99
1.01
1.02
1.02
1.01
9.1 9.0 8.9 8.8 8.7 8.6 8.5 8.4 8.3 8.2 8.1 8.0 7.9 7.8 7.7 7.6 7.5 7.4 7.3 7.2 7.1 7.0 6.9 6.8 6.7 6.6
f1 (ppm)

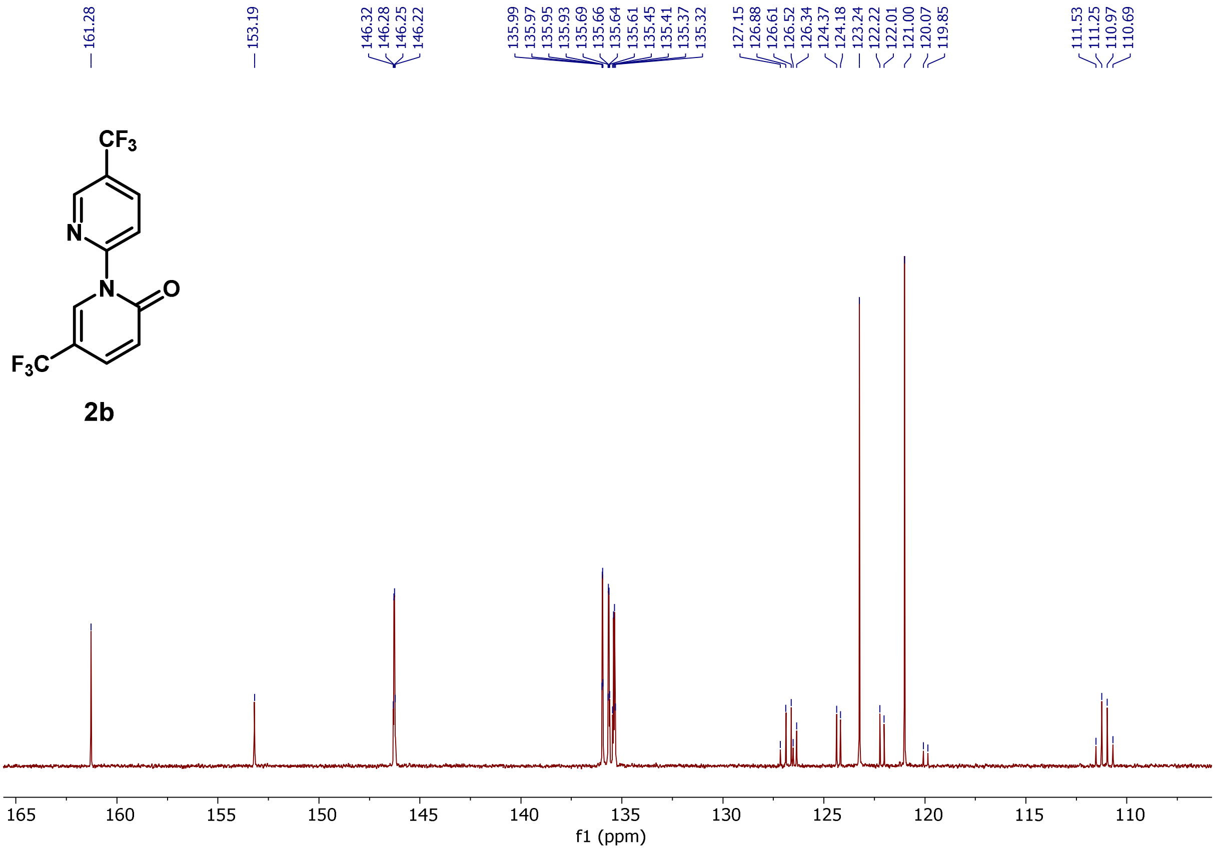
161.28
153.19
146.32
146.28
146.25
146.22
135.99
135.97
135.95
135.93
135.69
135.66
135.64
135.61
135.45
135.41
135.37
135.32
127.15
126.88
126.61
126.52
126.34
124.37
124.18
123.24
122.22
122.01
121.00
120.07
119.85
111.53
111.25
110.97
110.69
CF3
N
N
O
F3C
2b
165 160 155 150 145 140 135 130 125 120 115 110
f1 (ppm)

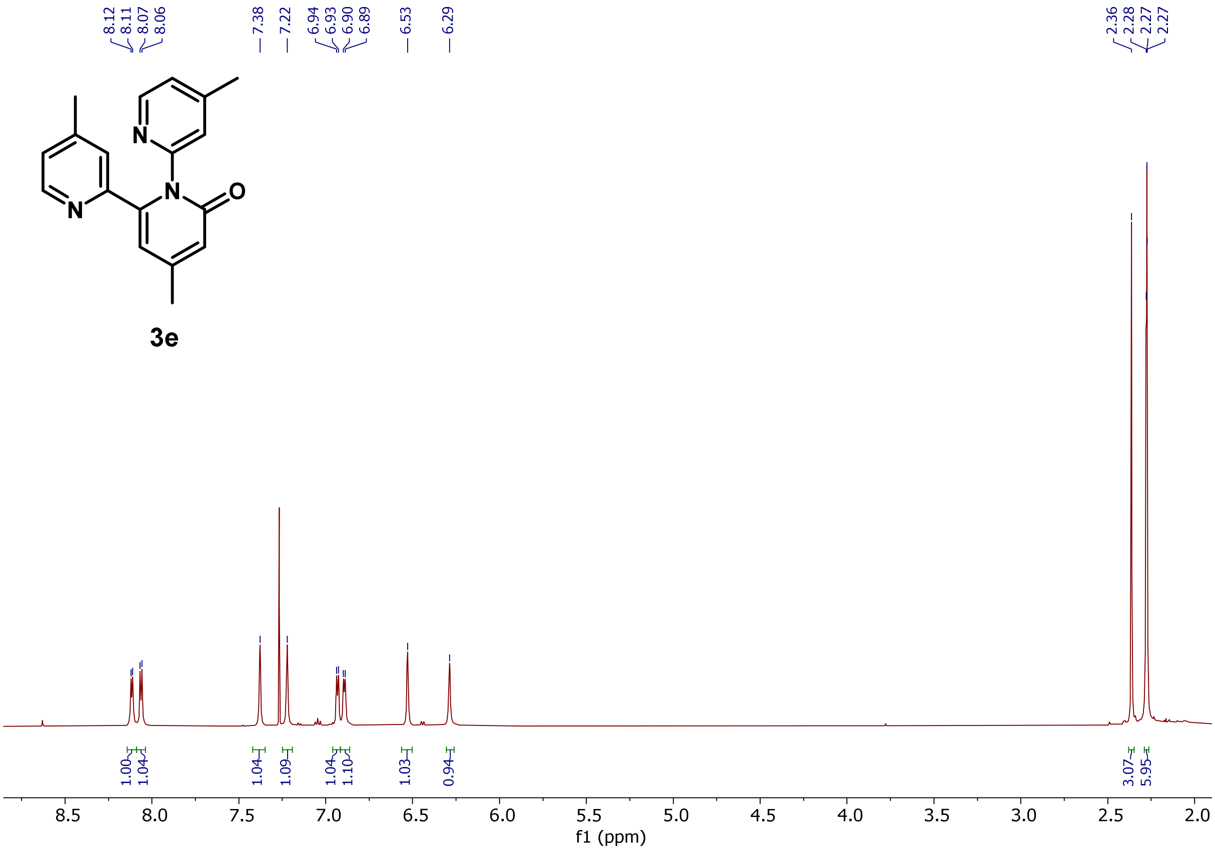
8.12
8.11
8.07
8.06
7.38
7.22
6.94
6.93
6.90
6.89
6.53
6.29
2.36
2.28
2.27
2.27
3e
1.00
1.04
1.04
1.09
1.04
1.10
1.03
0.94
3.07
5.95
8.5
8.0
7.5
7.0
6.5
6.0
5.5
5.0
4.5
4.0
3.5
3.0
2.5
2.0
f1 (ppm)

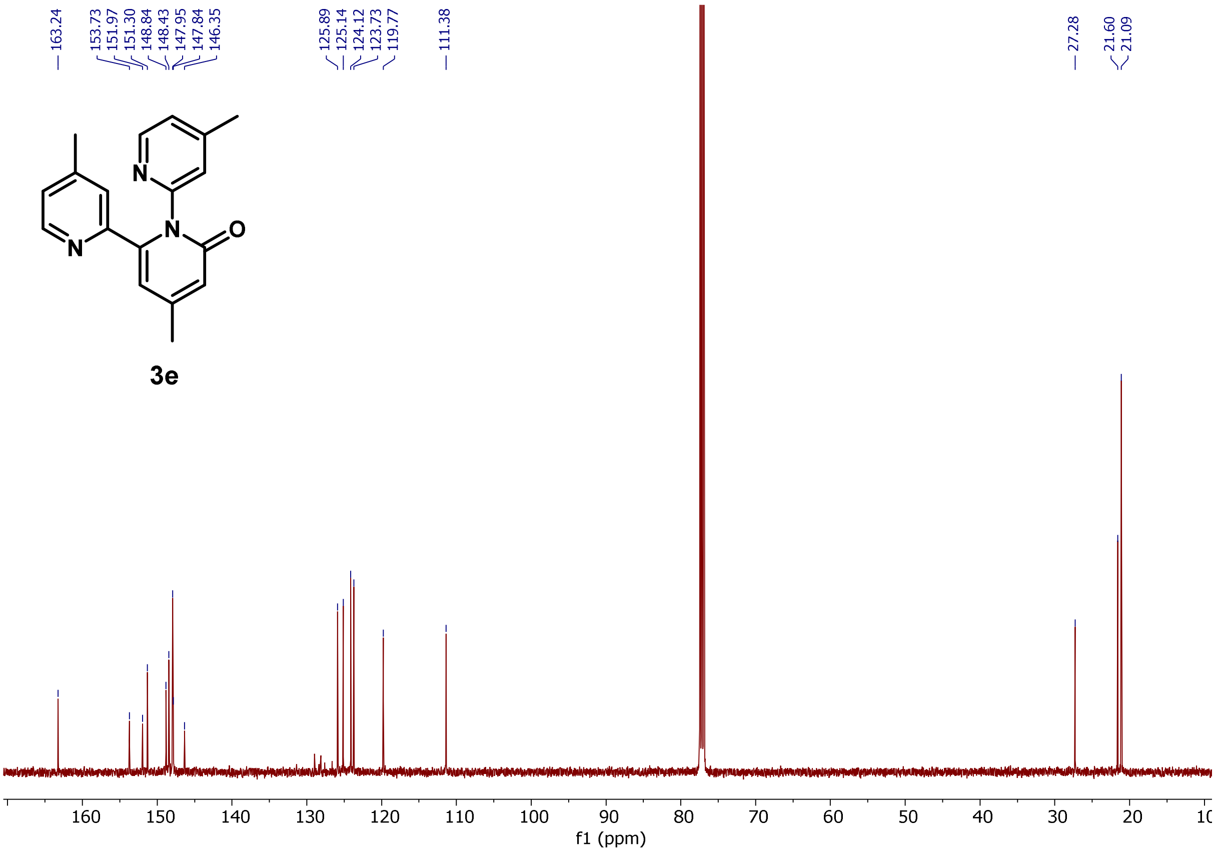
163.24
153.73
151.97
151.30
148.84
148.43
147.95
147.84
146.35
125.89
125.14
124.12
123.73
119.77
111.38
27.28
21.60
21.09
3e
160
150
140
130
120
110
100
90
80
70
60
50
40
30
20
f1 (ppm)

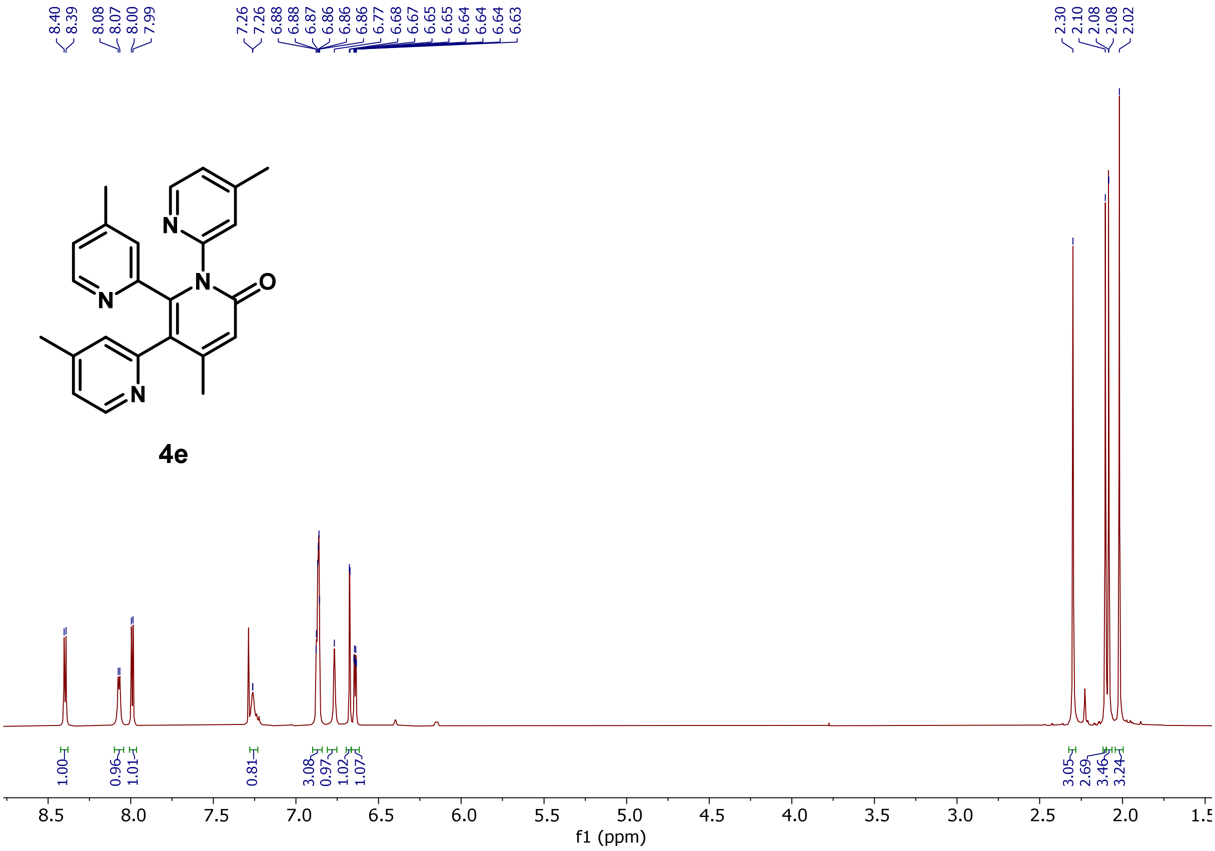
8.40
8.39
8.08
8.07
8.00
7.99
7.26
7.26
6.88
6.87
6.86
6.86
6.86
6.77
6.68
6.67
6.65
6.65
6.64
6.64
6.63
2.30
2.10
2.08
2.08
2.02
4e
1.00
0.96
1.01
0.81
3.08
0.97
1.02
1.07
3.05
2.69
3.46
3.24
f1 (ppm)

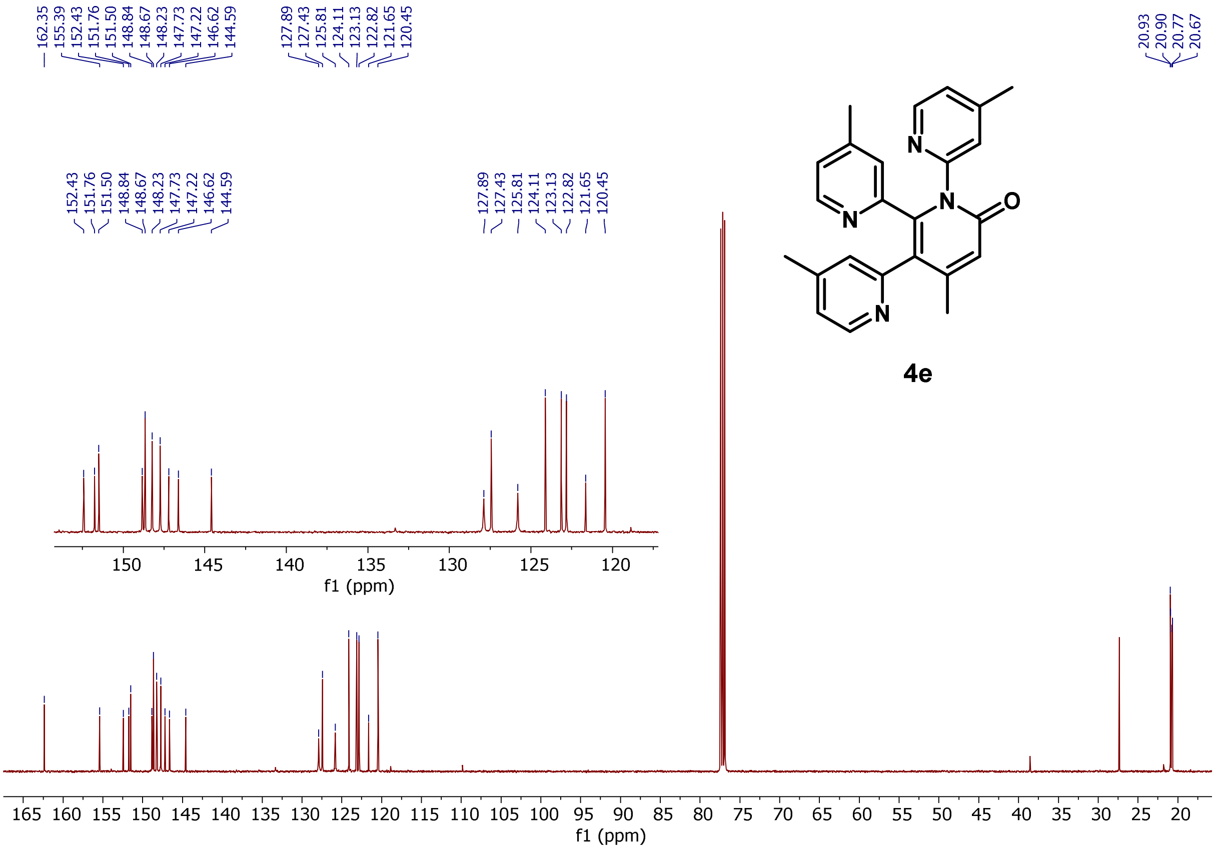
162.35
155.39
152.43
151.76
151.50
148.84
148.67
148.23
147.73
147.22
146.62
144.59
127.89
127.43
125.81
124.11
123.13
122.82
121.65
120.45
20.93
20.90
20.77
20.67
4e
f1 (ppm)

3c

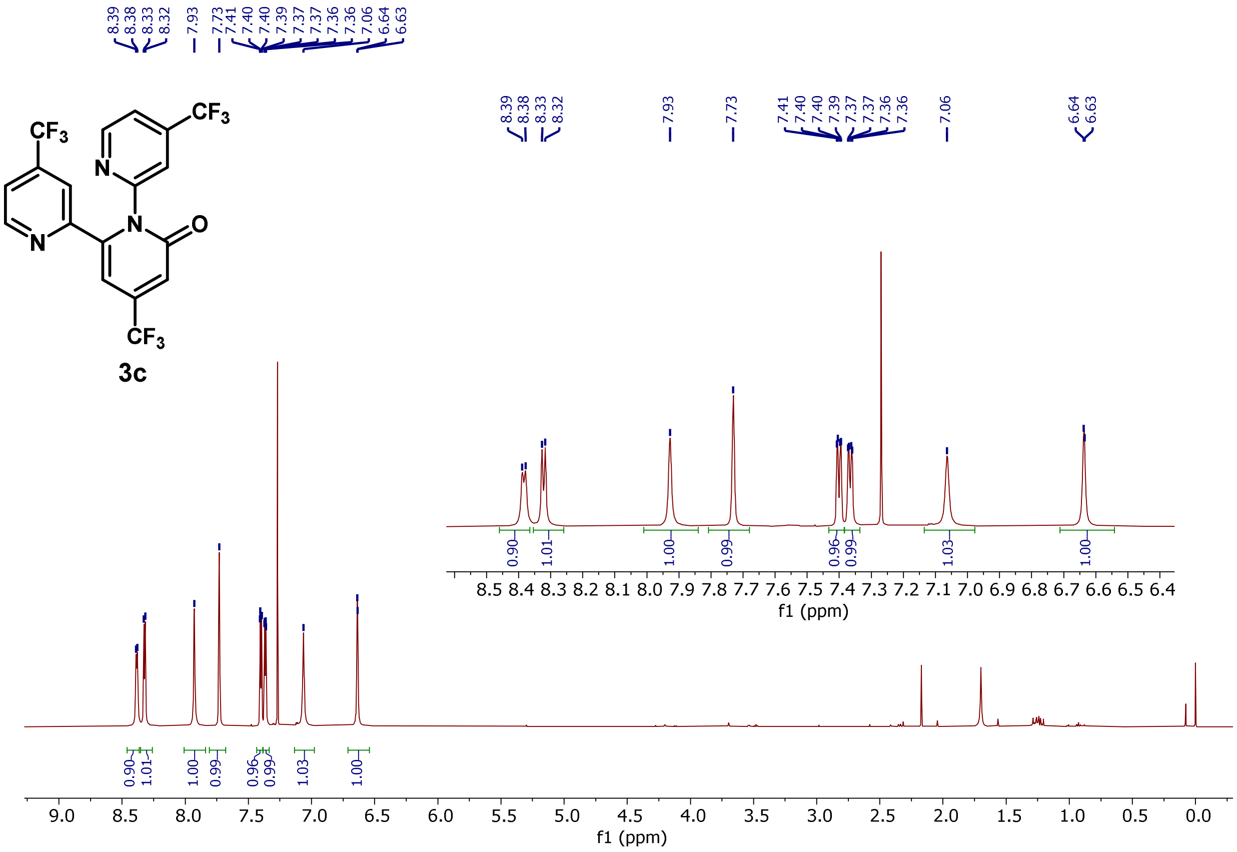
8.39
8.38
8.33
8.32
7.93
7.73
7.41
7.40
7.40
7.39
7.37
7.37
7.36
7.36
7.06
6.64
6.63
0.90
1.01
1.00
0.99
0.96
0.99
1.03
1.00
f1 (ppm)

3c

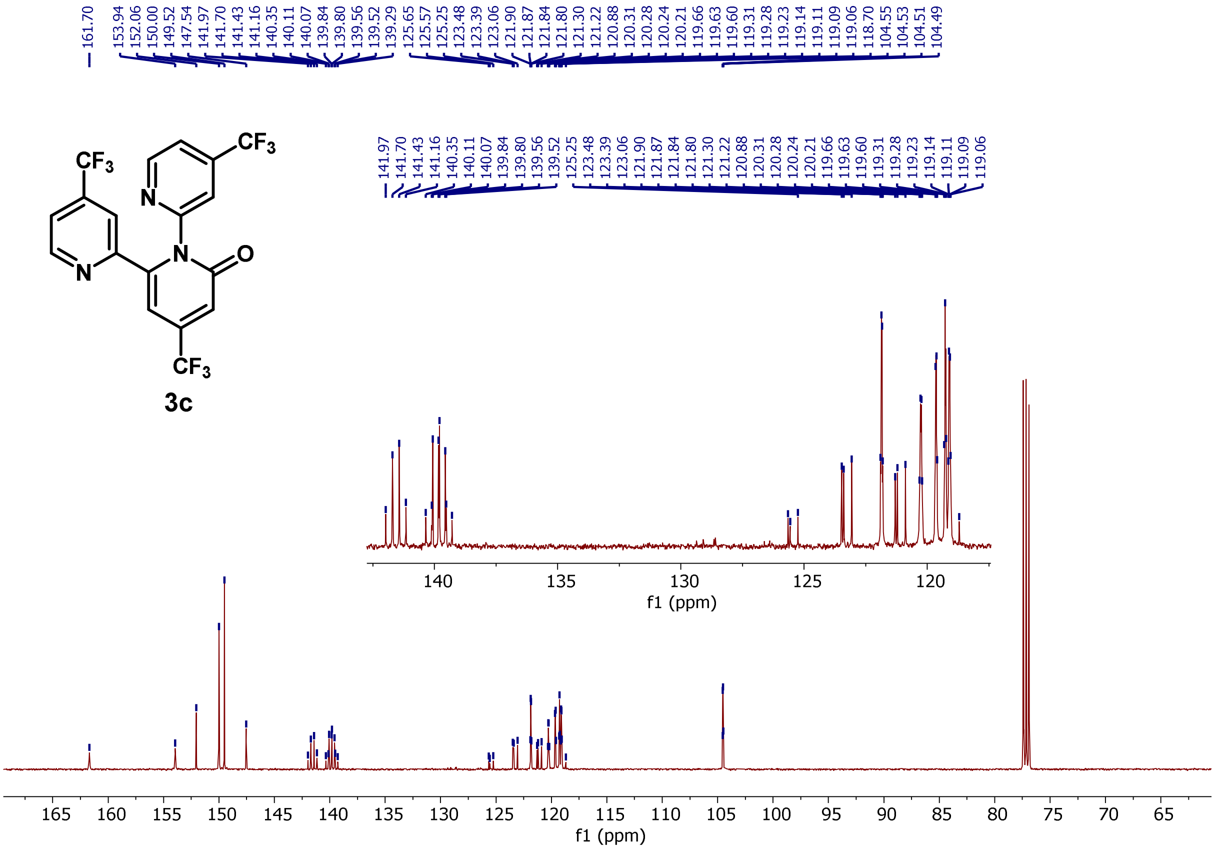
161.70
153.94
152.06
150.00
149.52
147.54
141.97
141.70
141.43
141.16
140.35
140.11
140.07
139.84
139.80
139.56
139.52
139.29
125.65
125.57
125.25
123.48
123.39
123.06
121.90
121.87
121.84
121.80
121.30
121.22
120.88
120.31
120.28
120.24
120.21
119.66
119.63
119.60
119.31
119.28
119.23
119.14
119.11
119.09
119.06
118.70
104.55
104.53
104.51
104.49
f1 (ppm)

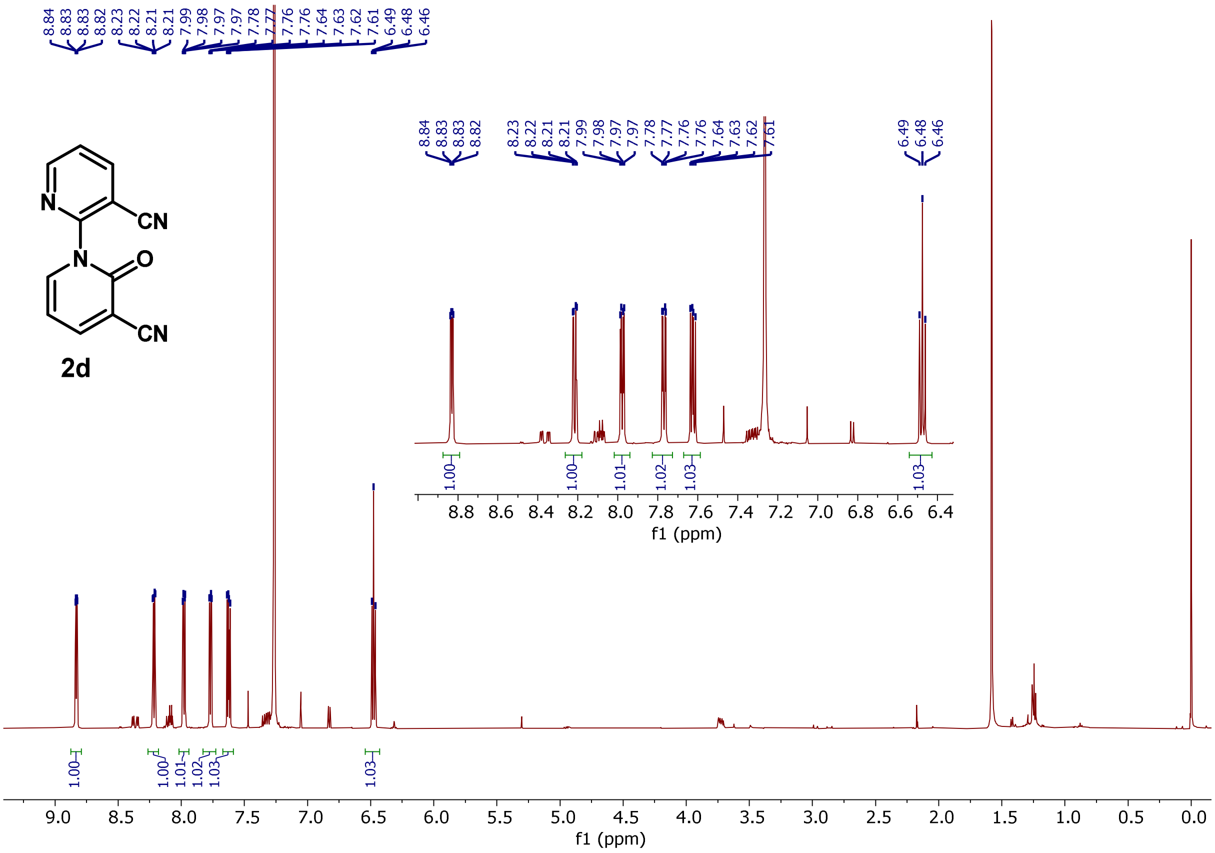
8.84 8.83 8.83 8.82 8.23 8.22 8.21 8.21 7.99 7.98 7.97 7.97 7.78 7.77 7.76 7.76 7.64 7.63 7.62 7.61 6.49 6.48 6.46
N
CN
N
O
CN
2d
1.00 1.00 1.01 1.02 1.03 1.03
f1 (ppm)

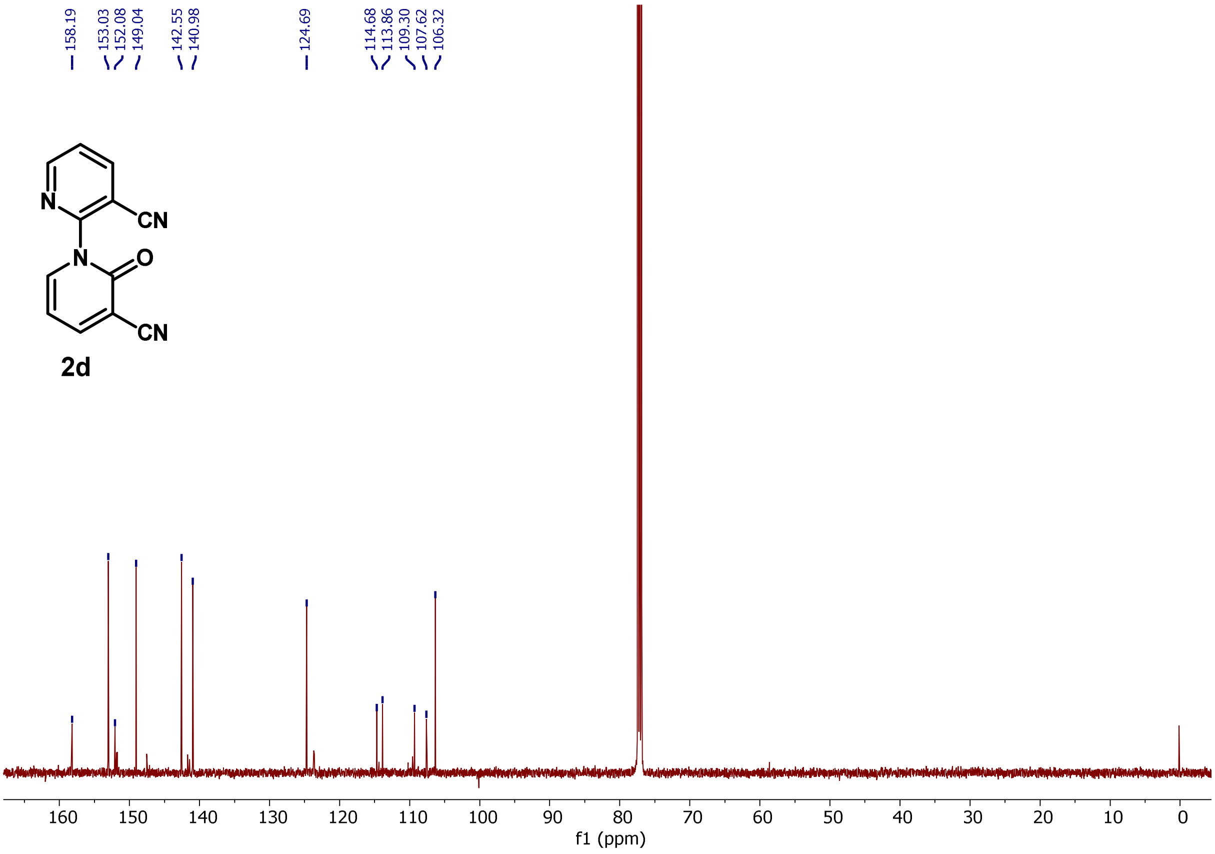
158.19 153.03 152.08 149.04 142.55 140.98 124.69 114.68 113.86 109.30 107.62 106.32
N
CN
N
O
CN
2d
f1 (ppm)

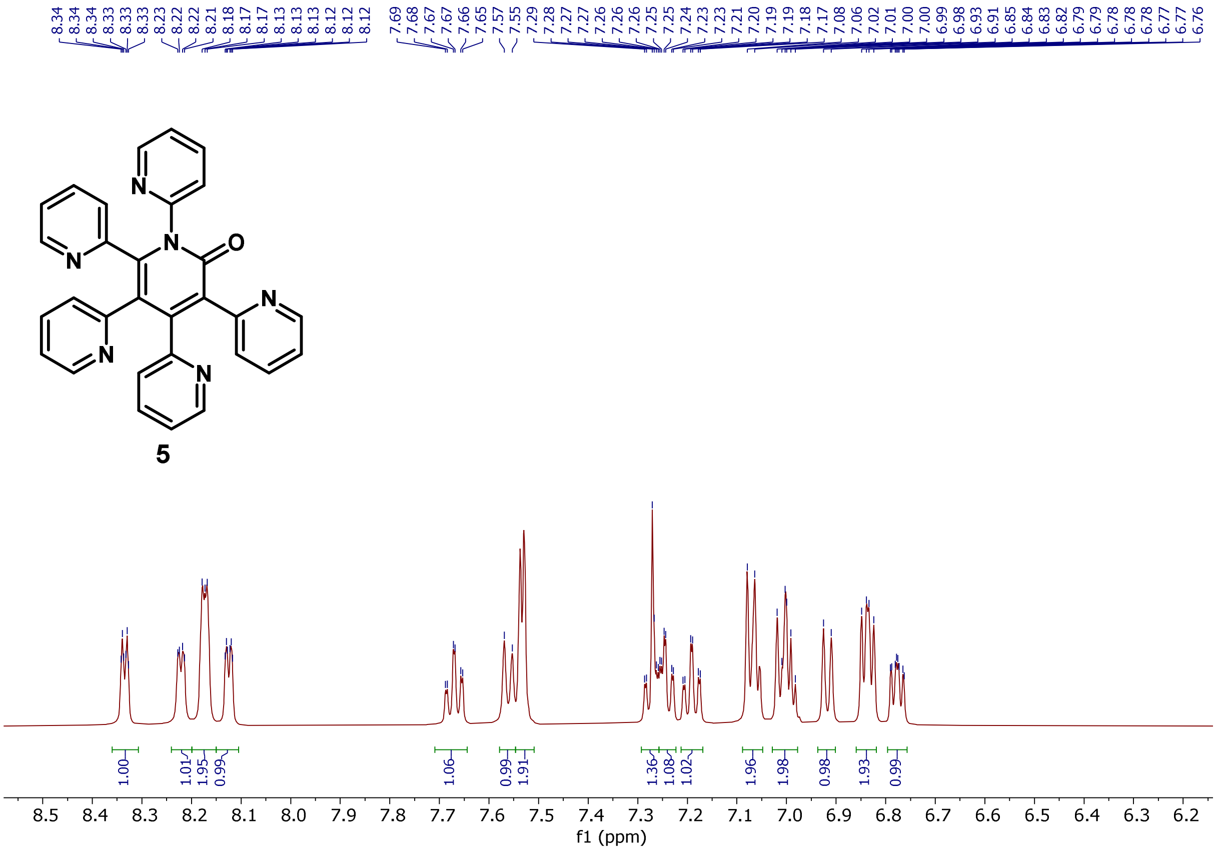
8.34 8.34 8.34 8.33 8.33 8.33 8.23 8.22 8.22 8.21 8.18 8.17 8.17 8.13 8.13 8.13 8.12 8.12 8.12 7.69 7.68 7.67 7.67 7.66 7.65 7.57 7.55 7.29 7.28 7.27 7.27 7.26 7.26 7.26 7.25 7.25 7.24 7.23 7.23 7.21 7.20 7.19 7.19 7.18 7.17 7.08 7.06 7.02 7.01 7.00 7.00 6.99 6.98 6.93 6.91 6.85 6.84 6.83 6.82 6.79 6.79 6.78 6.78 6.78 6.77 6.77 6.76
N
N
O
N
N
N
N
5
1.00
1.01
1.95
0.99
1.06
0.99
1.91
1.36
1.08
1.02
1.96
1.98
0.98
1.93
0.99
8.5 8.4 8.3 8.2 8.1 8.0 7.9 7.8 7.7 7.6 7.5 7.4 7.3 7.2 7.1 7.0 6.9 6.8 6.7 6.6 6.5 6.4 6.3 6.2
f1 (ppm)

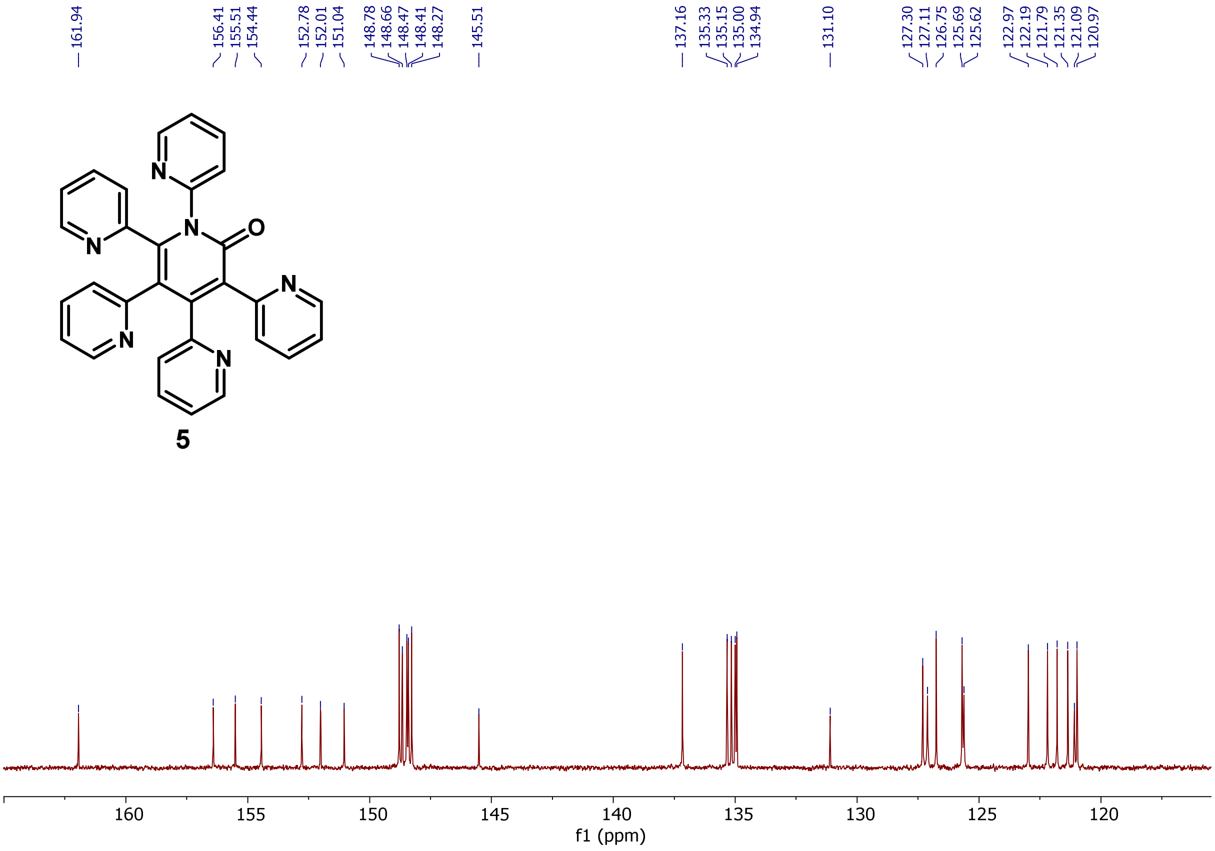
161.94
156.41 155.51 154.44
152.78 152.01 151.04
148.78 148.66 148.47 148.41 148.27
145.51
137.16
135.33 135.15 135.00 134.94
131.10
127.30 127.11 126.75 125.69 125.62
122.97 122.19 121.79 121.35 121.09 120.97
N
N
O
N
N
N
N
5
160 155 150 145 140 135 130 125 120
f1 (ppm)

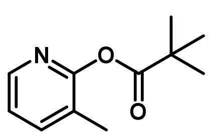

6

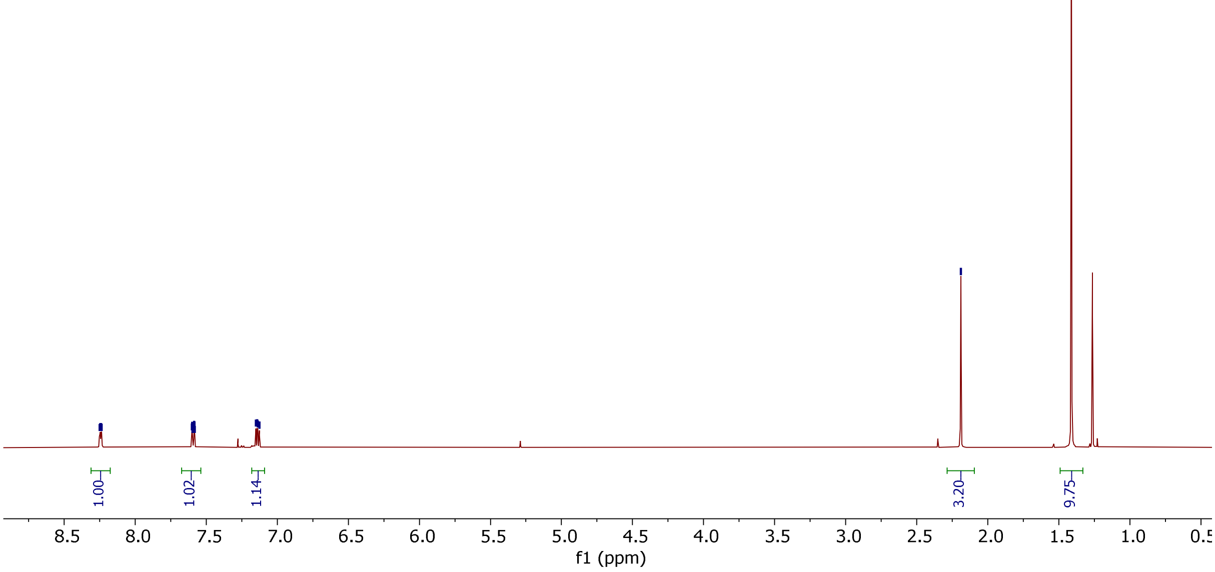
8.25 8.25 8.24 8.24 7.60 7.60 7.60 7.60 7.59 7.58 7.58 7.58 7.15 7.14 7.14 7.13
2.19
1.41
1.00 1.02 1.14 3.20 9.75
8.5 8.0 7.5 7.0 6.5 6.0 5.5 5.0 4.5 4.0 3.5 3.0 2.5 2.0 1.5 1.0
f1 (ppm)

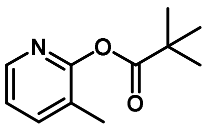

6

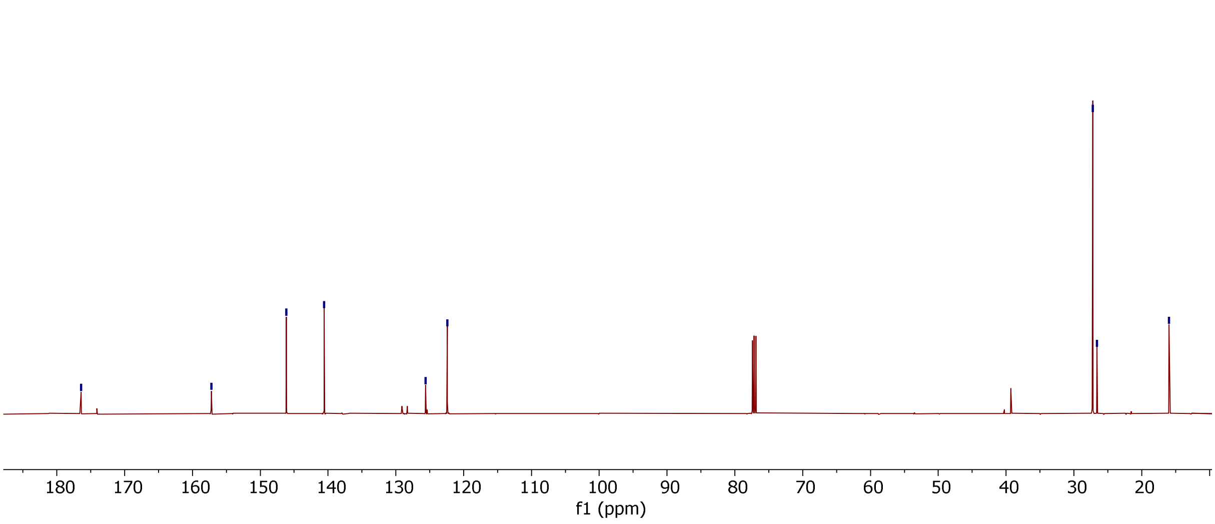
176.43
157.22
146.13
140.56
125.64
122.40
27.22
26.58
15.96
180 170 160 150 140 130 120 110 100 90 80 70 60 50 40 30 20
f1 (ppm)

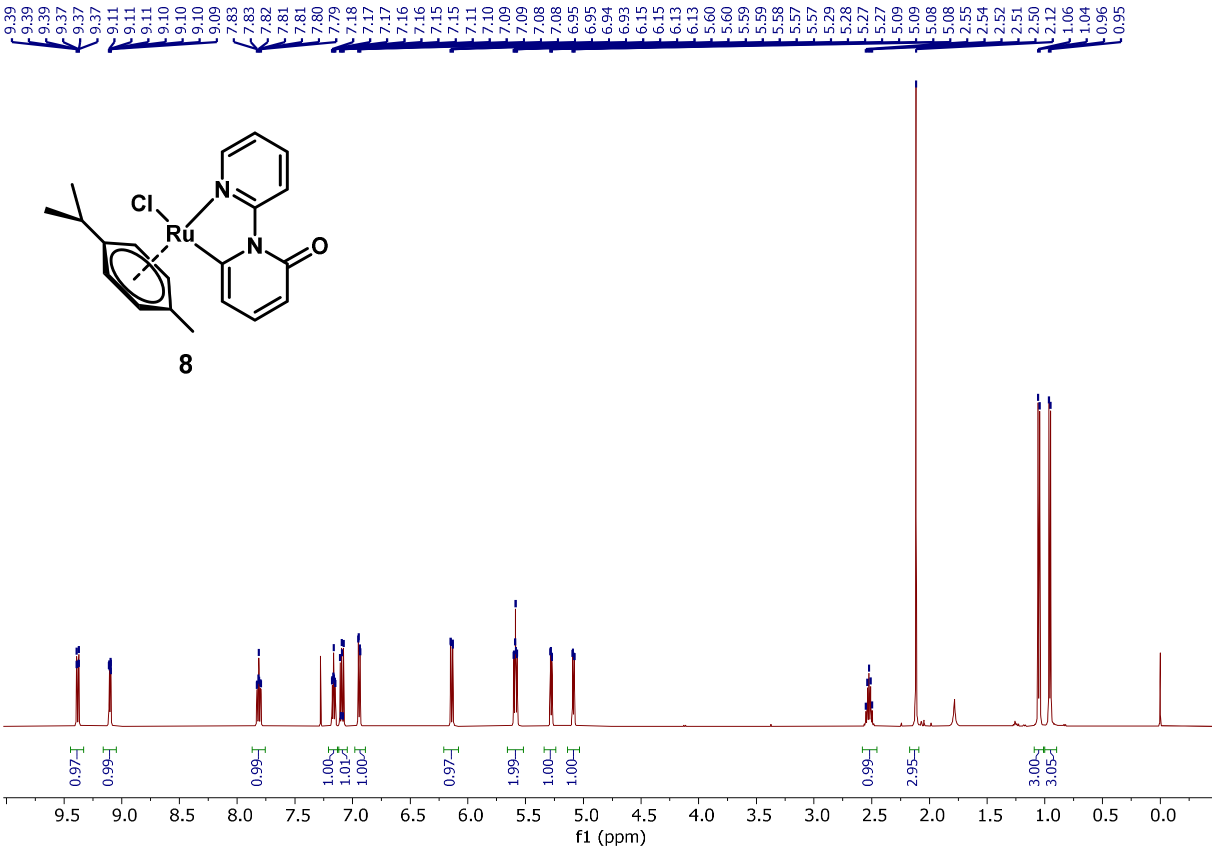
9.39 9.39 9.39 9.37 9.37 9.37 9.11 9.11 9.11 9.10 9.10 9.09 7.83 7.83 7.82 7.81 7.81 7.80 7.79 7.18 7.17 7.17 7.16 7.16 7.15 7.15 7.11 7.10 7.09 7.09 7.08 7.08 6.95 6.95 6.94 6.93 6.15 6.15 6.13 6.13 5.60 5.60 5.59 5.59 5.58 5.57 5.57 5.29 5.28 5.27 5.27 5.09 5.09 5.08 5.08 2.55 2.54 2.52 2.51 2.50 2.12 1.06 1.04 0.96 0.95
Cl
Ru
N
N
O
8
0.97 0.99 0.99 1.00 1.01 1.00 0.97 1.99 1.00 1.00 0.99 2.95 3.00 3.05
9.5 9.0 8.5 8.0 7.5 7.0 6.5 6.0 5.5 5.0 4.5 4.0 3.5 3.0 2.5 2.0 1.5 1.0 0.5 0.0
f1 (ppm)

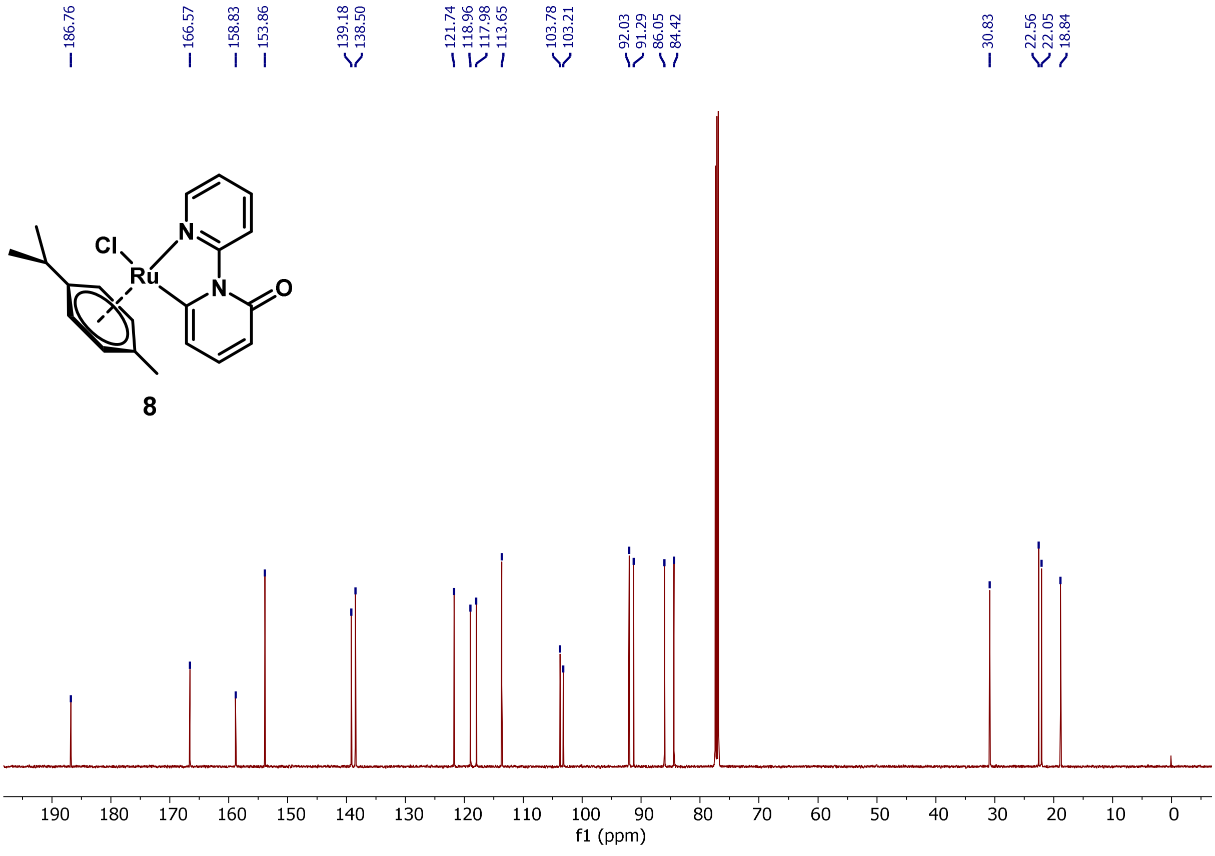
186.76 166.57 158.83 153.86 139.18 138.50 121.74 118.96 117.98 113.65 103.78 103.21 92.03 91.29 86.05 84.42 30.83 22.56 22.05 18.84
Cl
Ru
N
N
O
8
190 180 170 160 150 140 130 120 110 100 90 80 70 60 50 40 30 20 10 0
f1 (ppm)

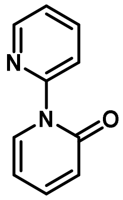

2f

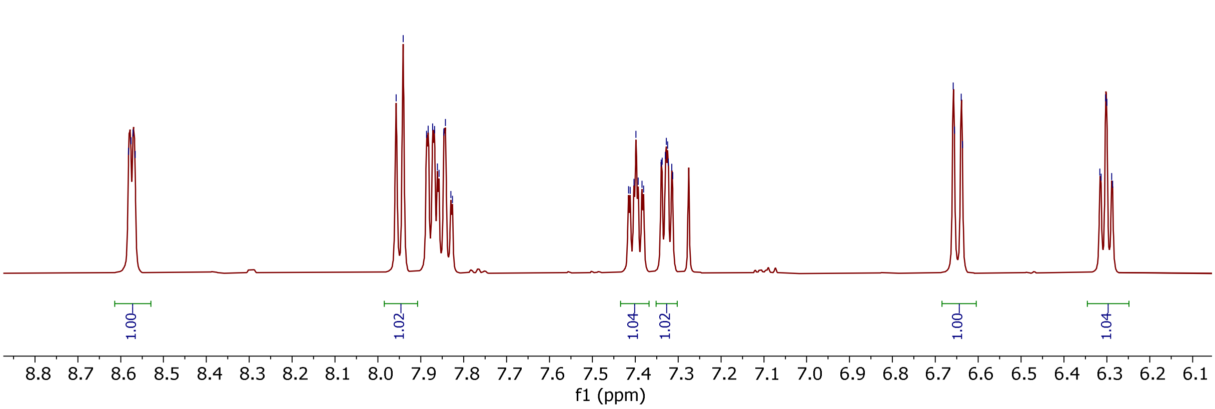

Supplement: Supplementary file 1 [file molecules-29-04418-s001.zip › molecules-3195522-supplementary.pdf]
